# Castration‐resistant prostate cancer cells are addicted to the high activity of cyclin‐dependent kinase 2

**DOI:** 10.1002/1878-0261.70319

**Published:** 2026-09-02

**Authors:** Joyeeta Chatterjee, Alessia Marin, Shivani Yalala, Harri M. Itkonen

**Affiliations:** ^1^ Department of Clinical Molecular Biology, EpiGen Institute of Clinical Medicine, University of Oslo Norway; ^2^ Department of Biochemistry and Developmental Biology, Faculty of Medicine University of Helsinki Finland; ^3^ EpiGen, Medical Division Akershus University Hospital Lørenskog Norway

**Keywords:** androgen receptor, cell cycle, cyclin‐dependent kinase 2, prostate cancer

## Abstract

Cyclin‐dependent kinases (CDK) drive the progression through the cell cycle and thereby form classical targets for cancer therapy. Targeting androgen receptor (AR) is effective against prostate cancer (PC), but frequently leads to development of incurable castration‐resistant PC (CRPC). We show that emergence of CRPC is associated with significant upregulation of CDK2 cyclins and downregulation of CDK4/6 cyclins. This rewiring renders CRPC cells dependent on the high CDK2 activity, and CDK2‐specific inhibitors efficiently decrease proliferation of PC and CRPC cells. We develop a CDK2 inhibitor‐resistant CRPC model and perform a rational compound screen to assess which of the currently used anti‐CRPC treatments could augment CDK2 therapy‐response. This dual approach revealed that when our CRPC model acquired resistance to CDK2 inhibition, it increased AR signaling, and the combination of CDK2 inhibition with anti‐androgens induces synergistic antiproliferative effects on CRPC cells. Importantly, CDK2 inhibitors also show synergistic interaction with cell cycle poisons such as Docetaxel and Cisplatin, and combinatorial effects are also seen with radiotherapy, thereby offering multiple combinations against CRPC. In brief, we propose that CDK2 inhibition provides a rational basis for combinatorial therapy against CRPC.

## Graphical abstract

We show that emergence of castration‐resistant prostate (CRPC) is associated with significant upregulation of cyclins that positively regulate cyclin‐dependent kinase 2 (CDK2) and concomitant downregulation of CDK4 cyclins. This renders CRPC cells dependent on the high activity of CDK2, and CDK2 inhibitors synergistically sensitize CRPC cells to both targeted and cytotoxic anti‐CRPC therapies.

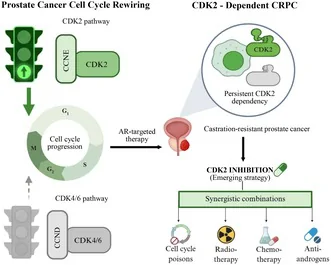

**Abbreviations**
ADT: androgen deprivation therapy
AR: androgen receptor
BSA: bovine serum albumin
CCNA: cyclin A
CCNB: cyclin B
CCND: cyclin D
CCNE: cyclin E
CDK: cyclin‐dependent kinase
CRPC: castration‐resistant prostate cancer
EDTA: ethylenediaminetetraacetic acid
EGTA: ethylene glycol‐bis(β‐aminoethyl ether)‐N,N,N′,N′‐tetraacetic acid
GAPDH: glyceraldehyde 3‐phosphate dehydrogenase
KLK3: kallikrein related peptidase 3
mTOR: mechanistic target of rapamycin
p53BP1: p53‐binding protein 1
PBS: phosphate‐buffered saline
PC: prostate cancer
RB1: Retinoblastoma protein 1

## 1 Introduction

Prostate cancer (PC) is the most frequently diagnosed cancer and a leading cause of cancer‐related mortality among men worldwide [1]. Initially, PC is an androgen‐dependent disease and can be controlled by androgen deprivation therapy (ADT). ADT targets androgen receptor (AR) signaling, and it is, at least initially, effective in most patients; however, a significant fraction develops castration‐resistant PC (CRPC) [2, 3, 4]. CRPC is characterized by sustained tumor growth despite castrate levels of circulating androgens, and it is associated with poor prognosis. At this point, the disease is currently incurable, but the so‐called next‐generation AR‐targeted agents, chemotherapy along with more experimental treatment strategies, provide extension to life expectancy. This is the state of the disease where new treatments are needed.

All proliferating cells are dependent on the cyclin‐dependent kinases (CDKs) that regulate progression through the cell cycle, which consists of two principal events: DNA replication and cell division [5, 6]. Cell cycle is controlled by the activity of cell cycle CDKs through cyclical expression of their cyclin partners. During G1 phase, mitogenic signals increase the expression of cyclin D, which binds to CDK4 and/or CDK6. The cyclin D – CDK4/6 complex mono‐phosphorylates Retinoblastoma protein 1 (RB1) protein, which weakens its interaction with the E2F transcription factors. In this situation, E2F activity rises modestly leading to upregulation of cyclin E. With the accumulation of cyclin E in the G1 phase, it binds to CDK2. The cyclin E – CDK2 complex inactivates RB1 completely through its hyper‐phosphorylation. This sustains the expression of the E2F‐driven genes required for transition from G1 to S phase and throughout the S Phase. CDK2 additionally forms complexes with cyclin E and cyclin A to stimulate replication initiation and to support S phase progression, respectively [7]. Subsequent accumulation of cyclin A/B – CDK1 complex controls mitotic entry. Cyclin A/B – CDK1 complex activates APC/CDC20 ubiquitin ligase, which promotes mitotic exit through targeted cyclin‐degradation, thereby assuring, in part, that the DNA is replicated only once within the S Phase [8]. In mammalian cells, CDK1 can drive progression through the cell cycle in the absence of CDK2, CDK4, and CDK6, which in part has motivated development of inhibitors against the three nonessential kinases that cancer cells can have acquired dependency of [6, 9].

In cancer, CDKs are hyper‐activated due to increased expression of CDKs themselves, cyclins and/or decreased expression of the endogenous CDK inhibitory proteins [10, 11]. This results in uncontrolled transition throughout the different cell cycle phases and may open cancer cell‐selective vulnerabilities. In breast cancer, the notion that Cyclin D1 is overexpressed in the tumor cells and also required for development of breast cancer has motivated clinical trials and subsequent approval of CDK4/6 inhibitors against breast cancer [12, 13, 14]. CDK4/6 inhibitors aim to arrest the cells to G1 phase of the cell cycle and suppress tumor proliferation, while sparing the normal noncancerous tissues, which is further accomplished by targeting cancer cell‐specific anomaly, dependency on the estrogen receptor. The success in breast cancer has motivated clinical trials also in PC; however, the CDK4/6 inhibitors have failed to control the disease, and their development has been largely discontinued (see, for example NCT05999968, NCT02905318, NCT05617885, and NCT04408924) [15, 16]. Given that breast cancer cells are dependent on CDK4/6 activity, it raises the possibility that PC cells are dependent on other cell cycle CDKs.

Increased expression of Cyclin E (CCNE) has been proposed as a biomarker for acquired dependency on CDK2, which can guide treatment selection for the CDK2‐targeted therapies [17]. BLU‐222 and Tagtociclib (PF‐07104091) are investigational inhibitors of CDK2 [18, 19, 20]. In *CCNE1*‐amplified endometrial cancer cells, BLU‐222 disrupts the RB‐signaling, leading to G1 cell cycle arrest [19]. Further preclinical *in vitro* and *in vivo* studies suggest that combining BLU‐222 with other therapies provides additional benefit in the *CCNE1*‐altered endometrial cancers [19]. Currently, Phase I/II clinical trials are evaluating Tagtociclib in ovarian cancer and nonsmall cell lung cancer to assess safety and early efficacy signals (NCT05262400, NCT04553133).

Here, we sought to establish if altered CDK2 signaling is associated with emergence of the CRPC phenotype, and if CDK2 inhibitors can control proliferation of CRPC cells. We show that development of metastatic CRPC results in significant upregulation of the E‐type cyclins and concomitant downregulation of the D‐type cyclins. CDK2 inhibition suppresses proliferation of CRPC cells but fails to eliminate them. We develop a CDK2 inhibitor‐resistant CRPC model and map potential combinatorial toxicities in multiple CRPC cell lines with clinically used CRPC therapies in combination with BLU‐222 and Tagtociclib. Our data reveal that resistance to CDK2 inhibition results in acquired dependency on CDK4/6 in our CDK2 inhibitor‐resistant model. In addition, resistant cells hyper‐activate AR signaling, and, accordingly, the combination of anti‐androgens with CDK2 inhibitors is effective against multiple CRPC cell lines. This study suggests the rational of further development of CDK2 inhibitors against CRPC.

## 2 Materials and methods

### 2.1 Cell culture, compounds, preparation of cell lysates and isolation of mRNA

LNCaP (Research Resource Identifiers, RRID: CVCL_0395), C4‐2 (RRID: CVCL_4782), 22RV1 (RRID: CVCL_1045), PC3 (RRID: CVCL_0035), DU145 (RRID: CVCL_0105), VCaP (RRID: CVCL_2235) and RWPE‐1 (RRID: CVCL_3791) cell lines were obtained from the American Tissue Culture Collection, while PNT1 (CVCL_4804) cells were obtained from Sigma. All, except for RWPE‐1 cells, were maintained in the RPMI media supplemented with 10% fetal bovine serum (FBS). RWPE‐1 cells were maintained in keratinocyte serum‐free media. After acquisition of the cell lines, a high number of freeze‐downs was prepared to ensure that cell lines remain stable for the duration of experiments in a new project. By doing this, we are confident that these cell lines remain the same as they were when purchased. Experiments were performed in mycoplasma‐free conditions. For androgen deprivation experiments, 10% charcoal‐stripped serum in phenol red‐free RPMI was used. In all of the experiments, cells were allowed to adhere to the plate for at least 1 day before starting the treatments. BLU‐222, Tagtociclib, Docetaxel, Cabazitaxel, Carboplatin, Cisplatin, Etoposide, Olaparib, Rucaparib, Everolimus, CX‐5461, Enzalutamide and Darolutamide were obtained from MedChemExpress. Cisplatin was solubilized into sterile PBS, while other compounds were solubilized into sterile DMSO. Further dilutions were prepared in DMSO to achieve appropriate final concentration in such a way that the DMSO concentration never exceeds 0.5% when given to cells. For radiation experiments, the cells were exposed to a single dose of radiation (4 Gy).

For cell lysate preparation, we followed a previous protocol and performed every step at 4 °C [21]. In brief, cells were washed once with PBS, lysed with RIPA buffer (10 mmol·L^−1^ Tris/HCl, pH 8.0, 1 mmol·L^−1^ EDTA, 0.5 mmol·L^−1^ EGTA, 1% Triton X‐100, 0.1% sodium‐deoxycholate, 0.1% SDS, 140 mmol·L^−1^ NaCl), and supplemented with freshly added protease and phosphatase inhibitors (MedChemExpress), incubated for 30 min, centrifuged at full speed for 10 min, and the supernatant was collected. The protein concentration was measured using the bicinchoninic acid (BCA) assay after the supernatant was collected. Antibodies were obtained from: ABclonal: p‐RB1 (AP0484) in dilution 1 : 1000, Cell Signaling Technologies: CDK2 (2546) in dilution 1 : 1000, Proteintech: p53 (60283‐2‐Ig) in dilution 1 : 2000, and Abcam: Actin (ab49900) in dilution 1 : 10 000. Horseradish peroxidase‐conjugated secondary antibodies against cognate species were used to identify primary antibodies (dilution 1 : 5000). Imaging was performed using the ChemiDoc Imaging System (Bio‐Rad) so that no region of the image is over‐saturated. Signal intensity was quantified using image lab software according to manufacturer's instructions (Bio‐Rad).

For knockdown experiments, transfection of Silencer® Select siRNAs against CDK2 was achieved using RNAiMax (siRNA catalog # 4427038, IDs: s204 and 206; Thermo Fisher Scientific). For the validation of knockdown, cell lysate was collected after 72 h of transfection for western blot analysis as described above, while for the colony‐formation experiments, the effects were evaluated after 7 days.

### 2.2 Proliferation assays and spheroid experiments

Cells were treated 1 day after the plating, and cell viability was assessed after 4 days of treatment using the CellTiter‐Glo® 2.0 assay (Promega) according to the manufacturer's instructions. Colony‐formation assay was performed as previously reported [22]. In brief, cells were allowed to grow and adhere for 24 h prior to treatment. After 1 week of treatment, the cells were washed with PBS buffer and fixed onto the plates using ice‐cold 70% methanol for 2 min, followed by 100% methanol for 10 min. Cells were allowed to dry completely before staining them with 0.05% crystal violet for 10 min. Excess stain was removed by washing thoroughly with distilled water. Representative images were taken of the plates, after which cells were de‐stained using 10% acetic acid for 15 min on a plate shaker, and the absorbance was measured at 590 nm.

For spheroid experiments, C4‐2 or 22RV1 cells were plated in Matrigel (catalog number: 354234; Corning,) and allowed to grow for 1 day before treatments. Spheroids were treated as indicated for 7 days, after which example images were obtained using a Zeiss Axio Vert.A1 Microscope. Finally, CellTiter‐Glo® 2.0 assay (Promega) was used to lyse the spheroids, and the luminescence signal was recorded. The data generated is from three biological replicates.

### 2.3 RT‐PCR and expression profiling

RNA was isolated post‐treatment using illustra RNAspin Mini RNA Isolation Kit (Cytiva) following the manufacturer's instructions. The cDNA used for qPCR was prepared using qScript cDNA Synthesis Kit (Quanta Biosciences). Amplification was carried out as follows: 10 min at 95 °C, 40 cycles of 30 s at 60 °C, and 30 s of extension. Primers are provided as a Fig. S1. To assure that only one sequence is amplified in every case, we performed the melting curve analysis.

### 2.4 Immunofluorescence

Cells were plated on glass slides and treated as indicated. For collection, media was removed and cells fixed with ice‐cold 100% methanol and stored in −20 °C. RNAse H was purchased from New England Biolabs and used according to the manufacturer's instructions prior to antibody staining. For staining, cells were washed with PBS three times, once with 5% BSA in PBS, blocked for 1 h with 5% BSA in PBS, primary antibodies added (Anti‐DNA–RNA Hybrid Antibody in dilution 1 : 50, clone S9.6, MERCK, MABE1095, p53BP1 in dilution 1 : 50, Thermo Fisher Scientific, 83809‐1‐RR) for 1‐hour incubation at room temperature, washing for 5 min each with 5% BSA in PBS was repeated thrice, secondary antibodies were added (Alexa Fluor™ 488 goat anti‐mouse IgG, Alexa Fluor™ 546 goat anti‐rabbit IgG; both in dilution 1 : 300) for 1‐hour incubation at room temperature, washing for 5 min each with PBS and finally glass slides were mounted with fluorescent mounting media (ProLong™ Glass Antifade Mountant with NucBlue™ Stain). Zeiss Axio Imager.Z2 upright epifluorescence wide‐field microscope was used for imaging. Immunofluorescence imaging was performed using a 63× oil‐immersion objective. Images were acquired for two independent replicate sample sets under identical imaging conditions. After acquisition, images were opened and analyzed using zeiss zen software. For quantification, three individual cells were selected from each replicate image. Each cell was treated as an individual measurement for signal quantification. Nuclear segmentations were done manually using the DAPI channel. These were then applied to the corresponding antibody channels to quantify the signal intensity of the target antibody staining. Quantified values from the individual cells were then used for downstream analysis.

### 2.5 Development of a resistant cell line

To generate CDK2 inhibition‐resistant cells, the parental C4‐2 cell line was cultured under BLU‐222 using a stepwise dose‐escalation strategy. Cells were initially exposed to 500 nm BLU‐222 for 2 months, followed by 2 months in 1000 nm, and finally, for an additional 2 months in 1500 nm compound. Media was replaced every 48–72 h.

### 2.6 Statistical analysis and plots

All experiments were performed at least in triplicates and data are presented with standard error of the mean. Statistical analyses and experimental data were performed and/or visualized in RStudio using standard, publicly available R packages, including tidyverse, ggplot2, ggpubr, and rstatix. For drug‐combination analyses, synergy scores were calculated using the synergyfinder r package [23]. No custom analysis scripts or software were used for the analyses in this manuscript.

Statistical comparisons were performed between two predefined groups, rather than across all treatment groups simultaneously, with *P* < 0.05 considered statistically significant. Paired two‐tailed Student's *t*‐tests were used when measurements were generated from the same independent experimental replicate, where treatment and control values shared replicate‐level experimental variability. For experiments in which the control condition was normalized to a fixed value of 1 or 100%, a one‐sample *t*‐test was used to evaluate whether treatment values were significantly different from the fixed normalized control value.

## 3 Results

### 3.1 Emergence of castration‐resistant prostate cancer results in increased dependency on CDK2

**Fig. 1:**
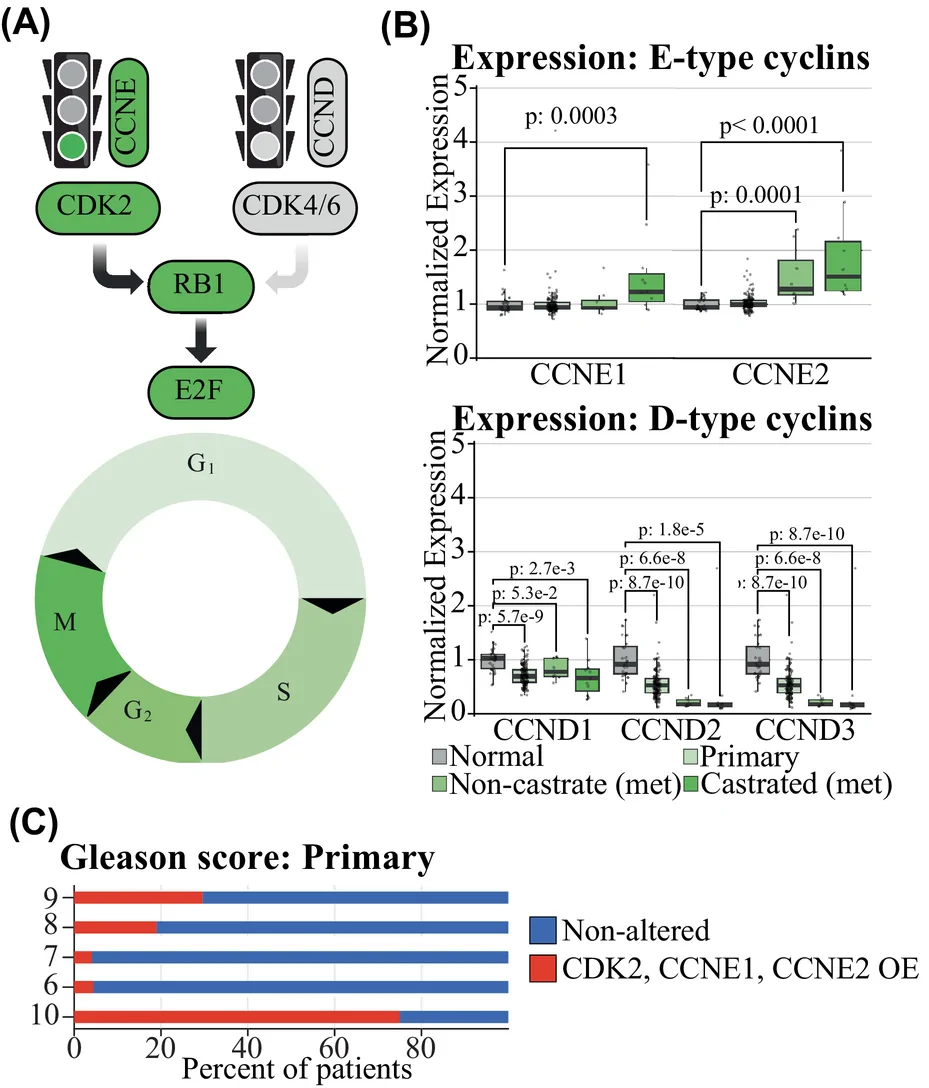
Development of aggressive, castration‐resistant prostate cancer is associated with increased expression of cyclin‐dependent kinase 2 (CDK2) cyclins. (A) Schematic representation of RB1‐E2F regulation during cell cycle (RB1: RB Transcriptional Corepressor 1, E2F: E2F Transcription Factor). Phosphorylation of RB1 releases the E2F transcription factor, which activates the genes required for cell cycle progression. (B) mRNA expression levels of E‐type cyclins (CCNE1 and CCNE2) and D‐type cyclins (CCND1, CCND2, and CCND3) were analyzed across normal prostate tissue, primary prostate cancer, metastatic prostate cancer, and castration‐resistant prostate cancer using patient datasets obtained from [24] accessed through betastasis.com. A total of 177 samples were analyzed, including 28 normal prostate samples, 130 primary prostate cancer samples, 8 metastasized non‐castrate prostate cancer samples and 11 metastasized castrate prostate cancer samples. Error bars represent the standard error of the mean (SEM) and an unpaired Mann–Whitney–Wilcoxon test was used to evaluate statistical significance (please, refer to figure for the values). (C) Gleason score distribution in prostate tumors with overexpression *CDK2* and/or *CCNE1* and/or *CCNE2* (exp > 2) accessed through the cBioPortal using the dataset “Prostate Adenocarcinoma (TCGA, Firehose Legacy)”. The expression threshold of > 2 was used as a stringent operational cutoff to define high expression of the mRNAs of interest. This cut‐off was chosen to reduce false‐positive classification of overexpression and to ensure that cases classified as CDK2‐high represented a clearly elevated‐expression group. A total of 491 samples with available CDK2 alteration and clinical annotation data were analyzed. Clinical variables were compared between CDK2‐altered and CDK2‐unaltered groups using the Wilcoxon test implemented in the cBioPortal. cBioPortal‐reported *q*‐values were used to account for multiple testing using the Benjamini–Hochberg false‐discovery‐rate method.

We wanted to understand if the development of CRPC is associated with altered expression of the key cyclins that control cell cycle progression. As detailed in the introduction, successful execution of the cell cycle depends on the sustained phosphorylation of RB1, which releases the E2F transcription factor to promote the cell cycle. CDK4/6 together with the D‐type cyclins (CCND) control decision to enter the cell cycle, while high activity of CDK2 brought about through the increased expression of CCNE is important to maintain the E2F activity throughout the cell cycle (Fig. 1A). After DNA synthesis is completed, the expression of CCNB1 increases, which stimulates CDK1 and completion of mitosis.

We evaluated the expression of these major cell cycle cyclins when the normal prostate tissue transforms initially to primary prostate cancer, followed by establishment of metastasis and eventual castration‐resistance. Strikingly, the expression of both the E‐type cyclins (CCNE1 and CCNE2) was significantly increased while D‐type cyclins (CCND1, CCND2, and CCND3) were significantly downregulated (Fig. 1B). The expression of the key regulator of mitosis, CDK1, and mitotic cyclins (CCNB1, CCNB2, CCNA1, CCNA2, and CCNA3) was also increased between normal prostate tissue and prostate cancer (Fig. S2A,B). In contrast, the expressions of CDK2, CDK4, and CDK6 did not reveal robust changes between the tissue types (Fig. S2B). Increased expression of CDK2, CCNE1 and CCNE2 was significantly associated with high Gleason Score (Fig. 1C). These data propose that prostate cancer cells, and particularly CRPC cells, are dependent on high activity of CDK2 to sustain the pro‐proliferative phenotype.

**Fig. 2:**
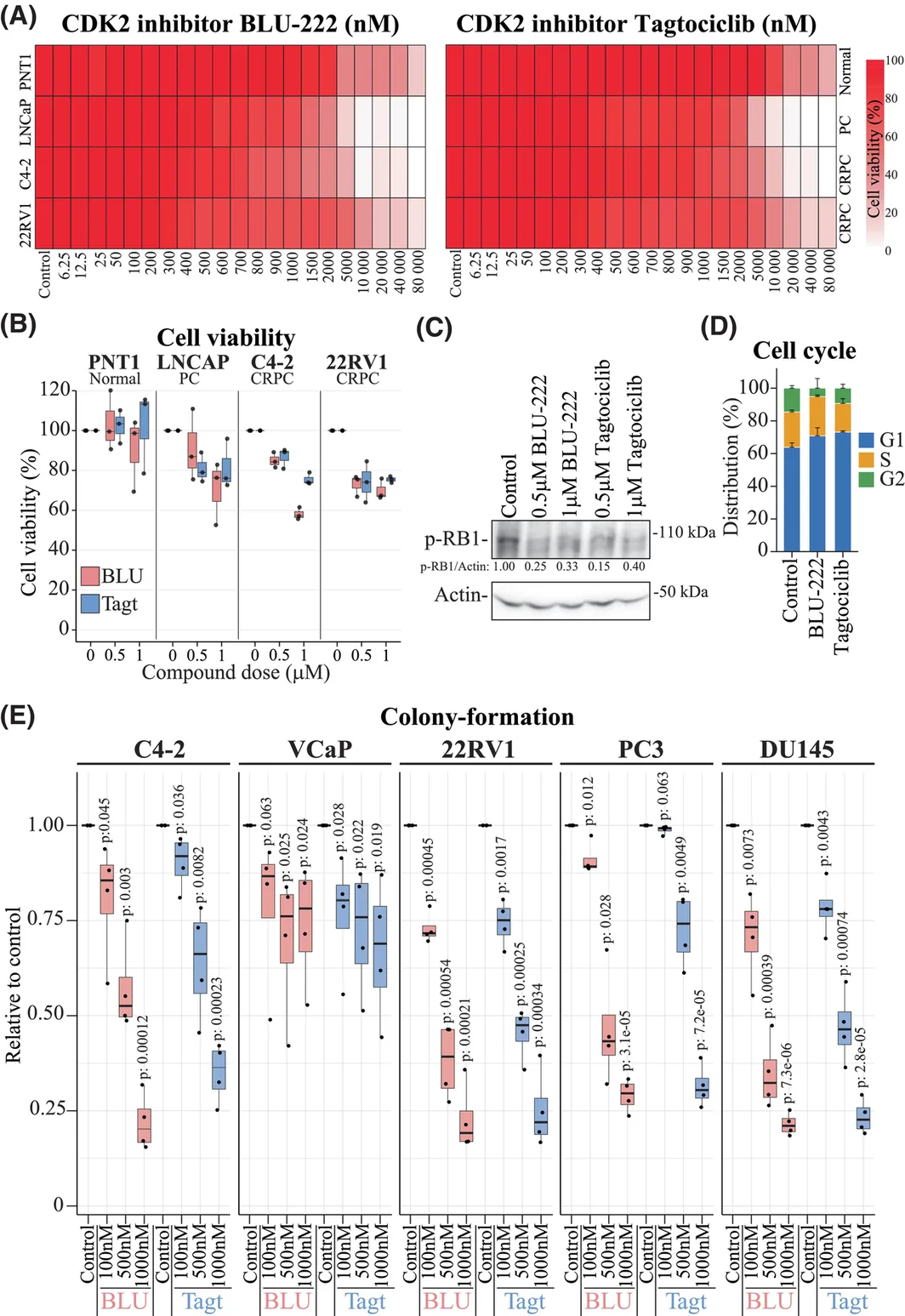
Prostate cancer cells are sensitive to CDK2 inhibition. (A and B) Cell viability after 4 days of treatment with BLU‐222 and Tagtociclib at the indicated concentrations (average of three biological replicates each with three technical replicates). Control samples were set to 100 and treatments were normalized to that. Error bars represent the standard error of mean (SEM). (C) Western blot‐based analysis of RB1‐phosphorylation after 24 h of indicated treatments in C4‐2 cell line. Data are from two biological replicate‐experiments. (D) Cell cycle‐distribution analysis after 3 days of treatment with 500 nm of the indicated CDK2 inhibitors in C4‐2 cells. Data are from three biological experiments and SEM is indicated. (E) Colony‐formation assay after 7‐day treatment (4 biological replicates for each cell lines). One‐sample *t*‐test was used to evaluate statistical significance, and error bars represent the SEM.

We moved on to probe if depletion of CDK2 activity is toxic to prostate cancer, CRPC, and a cell line derived from the normal prostate epithelia. First, we identified two structurally divergent CDK2 inhibitors, BLU‐222 and Tagtociclib [20, 25], and evaluated their efficacy against prostate cancer, CRPC, and normal cells. As expected, both compounds suppressed proliferation of prostate cancer and CRPC cell lines with lower doses than they affected normal prostate cells (Fig. 2A). We identified 500 nm dose as a dose that has practically no effect on normal cells but suppressed proliferation of cancer cells by at least 20% (Fig. 2A,B). Importantly, 500 nm BLU‐222 and Tagtociclib showed on‐target activity against CDK2 by decreasing phosphorylation of the RB1 protein by over threefold (Fig. 2C). This dose was sufficient to also cause a modest accumulation of cells into G1 phase (Fig. 2D). Prostate spheroid cultures using two CRPC models confirmed the significant and dose‐dependent antiproliferative effects (Fig. S3). To establish if expression of AR dictates cellular response to CDK2 inhibition, we performed colony‐formation assays in three AR‐positive CRPC cell lines (C4‐2, VCaP, and 22RV1) and two AR‐negative prostate cancer cell lines derived from metastatic sites (PC3 and DU145). These experiments revealed significant antiproliferative effects in all five models using 500 nm BLU‐222 or Tagtociclib, and this dose decreased the colony‐formation ability by 20–70% (Fig. 2E and Fig. S4). These data confirm that CDK2 inhibition can halt proliferation of both AR‐positive and AR‐negative prostate cancer cells. Finally, we confirmed that knockdown of CDK2 leads to an almost complete loss of the colony‐formation ability of CRPC cells (Fig. S5).

We have so far shown that the progression of prostate cancer to CRPC is associated with a transcriptional signature of CDK2‐dependency and confirmed the acquired dependency using two distinct compounds. However, while we detected robust antiproliferative effects, CRPC cells tolerated CDK2 inhibition reasonably well, and a better understanding of this should enable the design of rational combinatorial therapies.

### 3.2 Prolonged CDK2 inhibition transcriptionally upregulates CDK6‐CCND2 axis

**Fig. 3:**
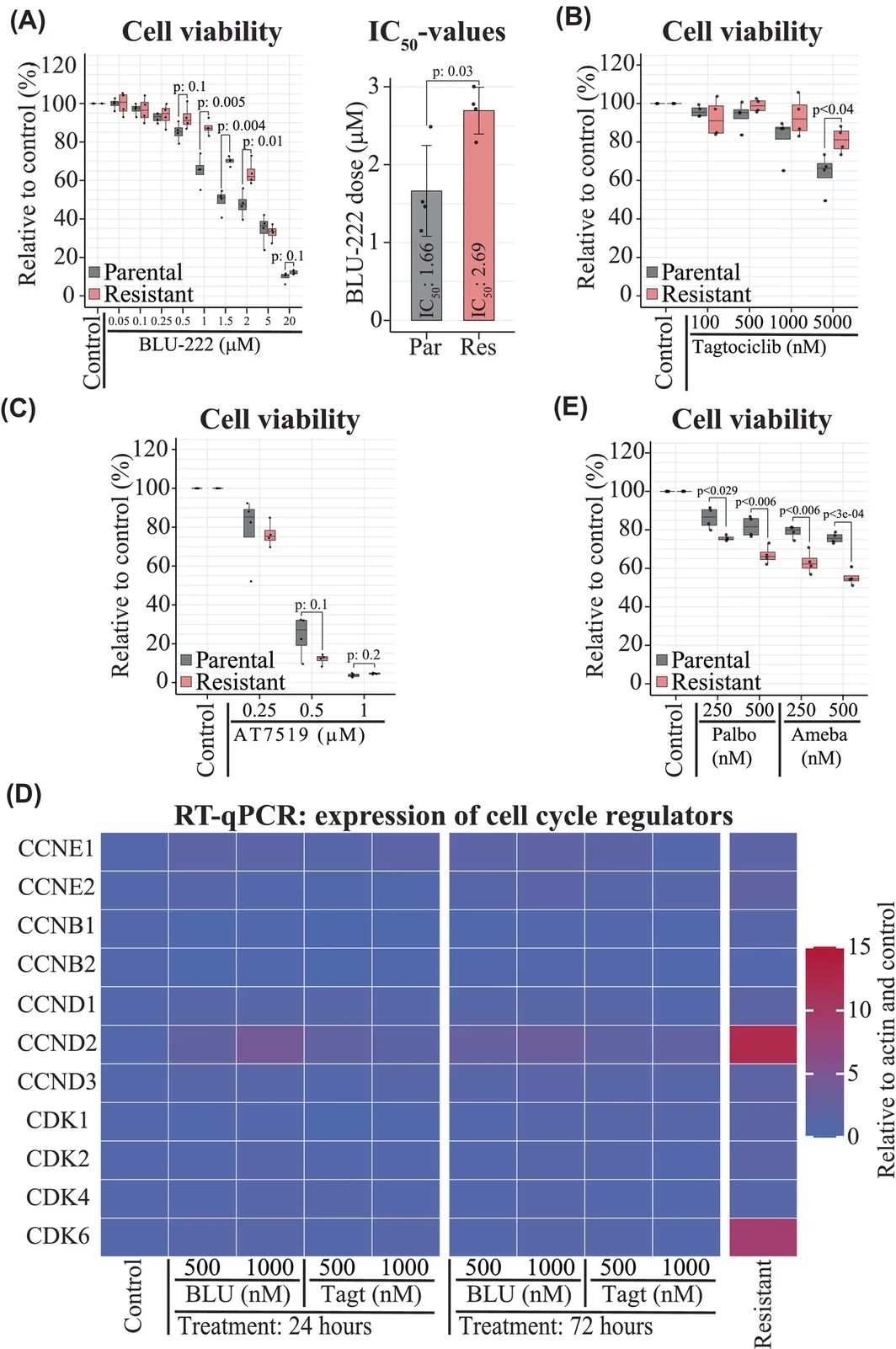
Resistance to CDK2 inhibition is associated with transcriptional rewiring to reinforce signaling through CDK6. (A) Parental and BLU‐222‐resistant C4‐2 cells were treated with BLU‐222 as indicated and cell viability was assessed after 4 days of treatment (four biological replicates, each 6 technical replicates and paired two‐tailed Student's *t*‐test was used to assess significance. graphpad prism was used to calculate IC_50_‐values. Error bars represent the SEM. (B, C) Parental and BLU‐222‐resistant C4‐2 cells were treated with the CDK2 inhibitor Tagtociclib or the pan‐CDK inhibitor AT7519 and viability assessed (the rest is the same as in 2A). (D) RT‐qPCR analysis of parental and BLU‐222 resistant C4‐2 cells after the indicated treatments. Data are from 3 biological replicates for the parental C4‐2 cells treated for 24 and 72 h, and 4 biological replicates for the comparison between parental and BLU‐222‐resistant C4‐2 cells (please, refer to Figs S6 and S7 for further details). (E) Parental and BLU‐222‐resistant C4‐2 cells were treated with the CDK4/6 inhibitor Abemaciclib and Palbociclib for 4 days and the rest is the same as in 2A. In every panel of this figure, we used either the parental C4‐2 cells (abbreviated as ‘Parental’ or ‘Par’) or the CDK2 inhibitor (BLU‐222) resistant cells (abbreviated as ‘Resistant’ or ‘Res’). Normalization mRNA is in every case actin. Please, refer to figure for the values on significance calculations.

We developed CDK2 inhibitor‐resistant cell line from the CRPC model C4‐2 to establish if acquisition of resistance is explained by transcriptional rewiring of the CDK/cyclin‐network. As expected, exposure of cells to CDK2 inhibitor suppressed proliferation and we therefore cultured the cells for an extended period of time in the presence of 500 nm BLU‐222, a dose that had no effect on the normal prostate cells but suppressed proliferation of PC and CRPC cells (Fig. 2B). After 2 months, when cells had started to proliferate again, we increased the dose to 1000 nm for 2 months, followed up by 2 months in the presence of 1500 nm BLU‐222. After this dose‐escalation, our novel cell line tolerated a significantly higher dose of the BLU‐222 compound (twofold, Fig. 3A). They also became cross‐resistant against another CDK2 inhibitor, Tagtociclib, but not against a pan‐CDK inhibitor AT7519 [26] (Fig. 3B,C).

We hypothesized that development of resistance occurs through transcriptional adaptation of the CDK/cyclin‐network to compensate for the lower CDK2 activity. To assess this, we exposed the parental C4‐2 cells to 500 nm BLU‐222 and 500 nm Tagtociclib for 24 and 72 h, isolated mRNA from untreated, treated, and resistant cells, and evaluated the levels of the key cell cycle cyclins. This experiment revealed a robust upregulation of CCND2 at 24 h (1.5‐ to 3‐fold), 72 h (1.5‐ to 3‐fold), and in the resistant cells (10‐fold) and also a modest increase in the CCNE1 and CCNE2 mRNAs (Fig. 3D and Fig. S6). At the same time, the expression of the mitotic cyclin CCNB decreased at both 24‐ and 72‐h and modestly increased in the resistant cells. These data propose that CRPC cells adapt to the decline in CDK2 activity by up‐regulating the CDK4/6 signaling. To further assess this, we evaluated the expression of the cell cycle kinases after short‐term and chronic CDK2 inhibition with BLU‐222 and Tagtociclib. Indeed, the expression of CDK6 was upregulated by ninefold in the resistant cells, but this effect was not detected after 24‐ or 72‐h treatments (Fig. 3D and Figs S6 and S7). In addition, we noted a modestly increased expression of all of the other cell cycle kinases that positively regulate progression through different stages of the cell cycle in the cells exposed to the CDK2 inhibitor chronically but not after 24‐ or 72‐h treatments (Fig. 3D and Figs S6 and S7).

Our transcriptional profiling data propose that acquisition of resistance to CDK2 inhibition causes acquired dependency on CDK6‐signaling. To directly assess this, we treated our parental and resistant cells with CDK4/6 inhibitors Palbociclib [27] and Abemaciclib [28]. Indeed, both CDK4/6 inhibitors had significantly stronger effect on the viability of the resistant cells when compared to the parental cells (Fig. 3E).

We have so far shown that CRPC cells have acquired dependency on CDK2, which can be overcome through transcriptional adaptation of the CDK‐/cyclin‐network. This adaptation involves robust upregulation of the Cyclin D2 and CDK6, while the cell cycle progression is initially halted as evidenced by the decrease in mitotic cyclins. Our resistant cell line is a single selected resistant population, and it was characterized only by using mRNA‐level analysis. In the future, it is of interest to develop additional CDK2 inhibitor‐resistant model systems and systematically assess protein‐level changes using an omics‐based approach and better understand if resistance is reversible. Nevertheless, our data are consistent with the prevailing observation from studies on CDK4/6 inhibition in breast cancer: cancer cells favor signaling through either CDK2 or CDK4/6 but have the ability to adapt if challenged by targeted inhibition of either CDK2 or CDK4/6 [29, 30, 31]. Our data show that inhibition of CDK2 activity induces changes in cell cycle cyclins and CDKs, and this should lead to replication stress, which we moved on to probe next.

### 3.3 CDK2 inhibition leads to formation of R‐loops and double‐strand DNA breaks

**Fig. 4:**
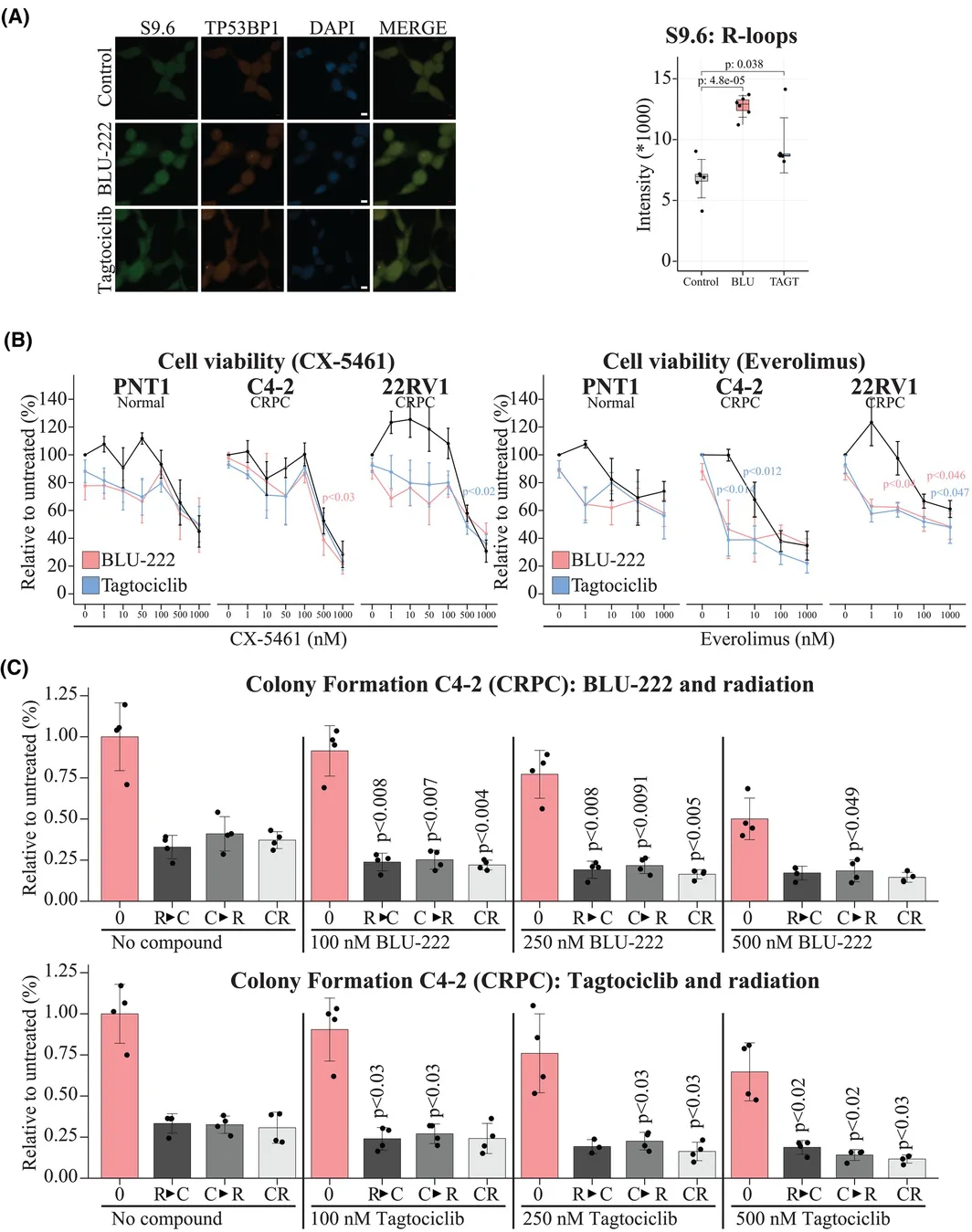
CDK2 inhibition causes replication stress and increases the efficacy of radiotherapy on prostate cancer cells. (A) C4‐2 cells were treated with BLU‐222 (500 nm) and Tagtociclib (500 nm) for 3 days, R‐loop accumulation was detected using immunofluorescence (the S9.6 antibody), and TP53BP1 was also detected (TP53BP1: Tumor Protein P53 Binding Protein 1). For quantification of signal intensity, three individual cells were selected from each of the two biological replicates, and each cell was treated as an individual measurement for signal quantification. Signal intensities with SEM are shown, and significance was assessed by paired two‐tailed Student's *t*‐test. Scale bar is 10 micrometers long. (B) CRPC (castration‐resistant prostate cancer) cell lines (C4‐2 and 22RV1) and normal prostate epithelial cells (PNT1) were treated for 4 days with CX‐5461 or Everolimus alone, or in combination with BLU‐222 (500 nm) or Tagtociclib (500 nm), cell viability assessed and data is the mean of four biological replicates (each biological replicate comprised of 2 technical replicates for PNT1 and 3 technical replicates for C4‐2 and 22RV1) with SEM. Paired two‐tailed Student's *t*‐test was used to evaluate statistical significance, which is indicated in comparison between CDK2 inhibitor‐alone and compound of interest‐alone to combination and is color‐coded according to CDK2 inhibitor used. (C) Colony‐formation assay. Cells were either not radiated or treated (0), first radiated and then treated after 1 day (R > C), first treated and then radiated after 1 day (C > R) or treated and radiated at the same time (CR). After 7 days, the colonies were detected (4 biological replicates with SEM). Paired two‐tailed Student's *t*‐test was used to evaluate statistical significance and the value reported is in comparison to both radiation‐alone and CDK2 inhibitor‐alone (whichever is higher, is reported). Please, refer to figure for the values on significance calculations.

CDK2 inhibition induced transcriptional changes to sustain cell cycle progression, and we hypothesized that these changes cause DNA replication stress. When transcription and replication machineries collide, this can lead to the generation of R‐loops, nucleic acid structures consisting of an RNA–DNA duplex and an unpaired DNA strand [32]. We assessed R‐loop formation as the first port‐of‐call to evaluate CDK2 inhibition‐induced replication stress. Indeed, treatment of C4‐2 cells with either of the CDK2 inhibitors, BLU‐222 or Tagtociclib, increased the R‐loop levels as determined using immunofluorescence (Fig. 4A). To confirm the specificity of this signal, we performed RNase H treatment (Fig. S8A). We hypothesized that the accumulation of R‐loops would lead to DNA damage. p53‐binding protein 1 (p53BP1) is recruited to double‐strand breaks [33], and we used it as a marker for DNA damage. Indeed, we noted a robust increase in the p53BP1 foci after treatment with both BLU‐222 or Tagtociclib (Fig. 3A) but did not detect persistent activation of DNA damage signaling as measured by H2AX phosphorylation at the same time point (Fig. S8B). Further inspection of the immunofluorescence data indicated that the R‐loops accumulate particularly in the densely packed areas in the nucleus, the areas responsible for the synthesis of ribosomal RNA.

We hypothesized that nucleolar stress and/or protein synthesis become points of vulnerability in combination with CDK2 inhibitors. For these experiments, we made use of CX‐5461, an inhibitor of RNA Pol I, the polymerase responsible for ribosomal RNA synthesis in the nucleolus [34] and Everolimus, an inhibitor of the mTOR kinase, major regulator of protein synthesis [35]. According to our hypothesis, both CX‐5461 and Everolimus in combination with CDK2 inhibitors decreased viability more than the single compounds in CRPC cells (C4‐2 and 22RV1, Fig. 4B). These combinations were less effective in a cell line derived from the normal prostate epithelia (PNT1, Fig. 4B). However, the effect was modest, and at best, additive, and a different combination strategy is therefore required to effectively target the CRPC cells, and we moved on to acquire further evidence on the DNA damage‐activation in response to CDK2 inhibition.

In support of activation of the DNA damage response, we detected robust accumulation of p53 as determined by western blotting after CDK2 knockdown or treatment with BLU‐222 and Tagtociclib (Fig. S8B,C). p53 functions as an important mediator of the cellular response to DNA damage [36]. However, we earlier detected a clear decrease in the proliferation of CRPC cells that either have wild‐type p53 (C4‐2) or a mutated one (VCaP, 22RV1, PC3, and DU145, Fig. 2A,B,D). Therefore, p53 itself is not necessary for the CDK2 inhibition‐induced antiproliferative effects.

We hypothesized that the antiproliferative effects of CDK2 inhibition could be significantly increased if DNA damage was induced. In the clinical setting, prostate cancer can be treated by radiotherapy, which effectively suppresses the tumor progression. However, radiation should arrest the cell cycle, which might decrease the efficacy of CDK2 inhibitors. To take this into account, we devised a four‐armed experimental strategy: single agent, first radiotherapy followed by CDK2 inhibitor, first CDK2 inhibitor followed by radiotherapy, and simultaneous treatment. Strikingly, all of these combinatorial treatment strategies significantly further suppressed the colony‐formation ability of both C4‐2 and 22RV1 when compared to single agent treatments (Fig. 4C and Fig. S9).

We have so far shown that CDK2 inhibition causes modest antiproliferative effects but prominent DNA replication stress, which significantly increases the efficacy of radiotherapy on CRPC cells. Next, we moved on to systematically probe if specific clinically utilized anti‐CRPC therapies could be combined with CDK2 inhibitors to cause synergistic antiproliferative effects.

### 3.4 CDK2 inhibition results in increased dependency on the high activity of androgen receptor

We established a library of compounds from currently used prostate cancer therapies to probe which would form the most effective combination with CDK2 inhibitors. The compounds in our library can be divided into two major groups: First, we have five cytotoxic compounds and second, more targeted compounds. In the first class, we included Etoposide, which targets topoisomerase II enzyme and thereby leads to defective resolution of negative and positive supercoils in DNA [37]. Carboplatin and Cisplatin cause inter‐ and intra‐strand DNA adducts, which interfere with DNA replication and transcription [38]. Docetaxel and Cabazitaxel target the assembly of microtubules into the mitotic spindle, thereby triggering G2/M arrest [39]. For more biomarker‐guided and disease‐specific therapies, we included Olaparib, Rucaparib, Enzalutamide, and Darolutamide. Olaparib and Rucaparib target PARP, which becomes a synthetic point of antiproliferative effects in cells that have mutations in the homologous recombination pathway [40], while Enzalutamide and Darolutamide are inhibitors of androgen receptor [41]. To identify combination(s) that are less toxic to normal cells, we included a cell line derived from normal prostate epithelia, PNT1, and two models of CRPC, C4‐2 and 22RV1. The screen was performed across four biological replicates and 2–3 technical replicates in each plate to assure robustness.

**Fig. 5:**
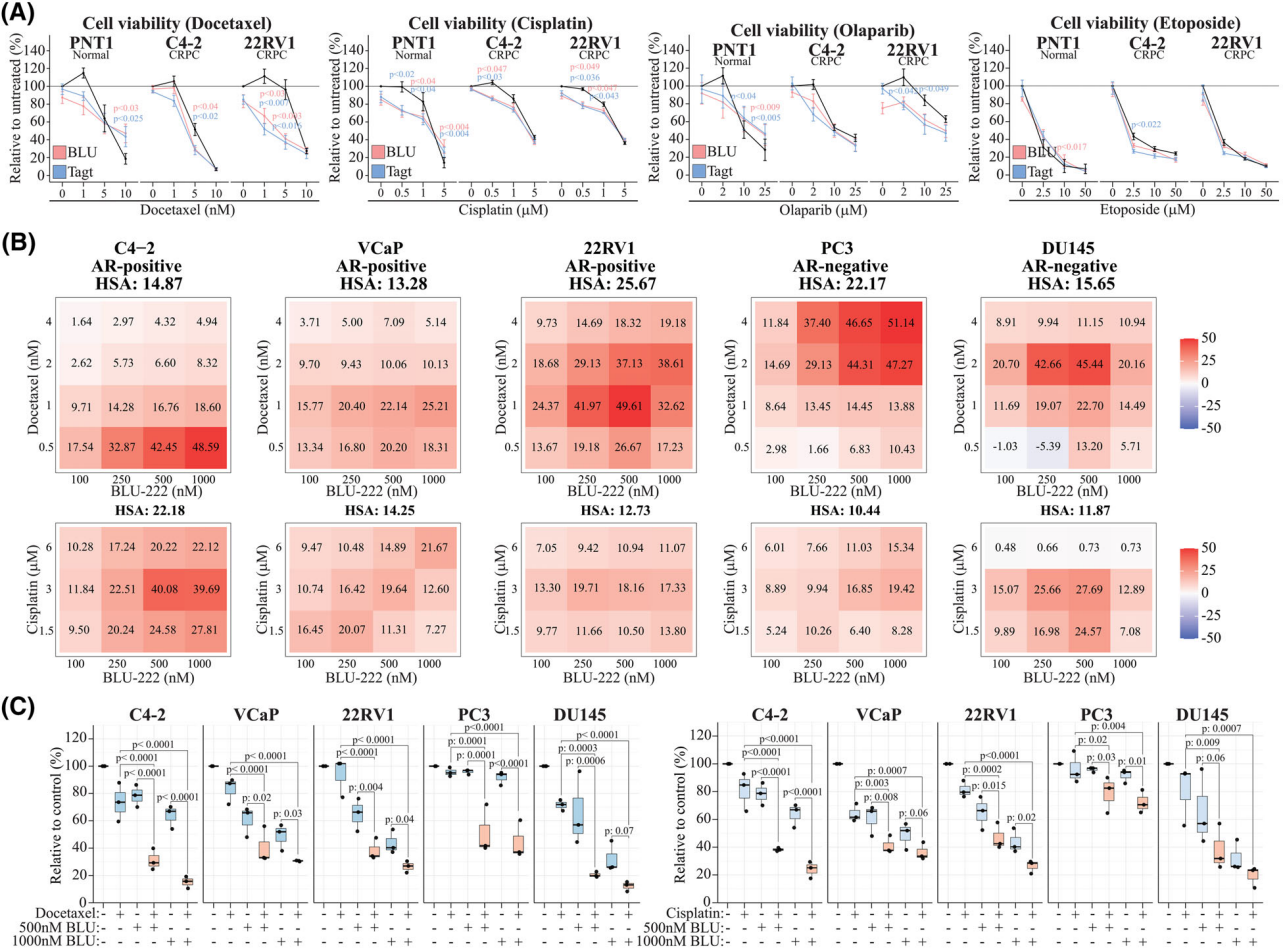
Targeted compound screen and subsequent validation reveal synergistic effects of CDK2 inhibition with Docetaxel and Cisplatin. (A) CRPC cell lines (C4‐2 and 22RV1) and cell line derived from normal prostate epithelia (PNT1) were treated for 4 days as indicated (BLU‐222: 500 nm and Tagtociclib: 500 nm), viability assay performed and data is the mean of four biological replicates with SEM (each biological replicate comprised of 2 technical replicates for PNT1 and 3 technical replicates for C4‐2 and 22RV1). Paired two‐tailed Student's *t*‐test was used to evaluate statistical significance, which is indicated in comparison between CDK2 inhibitor‐alone and compound of interest‐alone to combination and is color‐coded according to CDK2 inhibitor used. (B) Synergy maps of HSA scores between CDK2 inhibitor BLU‐222 and Docetaxel (above) or Cisplatin (below). Cells were treated for 7 days after which cell viability was assessed using CellTiterGlo and the data are from three biological replicates. (C) Cell viability assays after 7 days of treatment. Docetaxel dose is 0.5 nm for C4‐2, VCaP and 22RV1, and 2 nm for PC3 and DU145. Cisplatin dose was always 3 micromolar. The data are from three biological replicates with SEM. Paired two‐tailed Student's *t*‐test was used to evaluate statistical significance (please, refer to figure for the values on significance calculations).

Interestingly, the efficacy of CDK2 inhibitors was increased by cytotoxic compounds, while the targeted therapies had more variable efficacy (Fig. 5A and Fig. S10). In more detail, Docetaxel, Cabazitaxel, Cisplatin, and Carboplatin were toxic as single agents to all three model systems and CDK2 inhibition predominantly increased this toxicity. In contrast, PARP inhibitors and Etoposide were toxic at the single agent level to all of the cell line models but did not clearly enhance the efficacy of CDK2 inhibitors (Fig. 5A and Fig. S10). To better evaluate the combinatorial antiproliferative effects, we performed synergy calculations across multiple doses in five cell lines representing both AR‐positive (C4‐2, 22RV1, and VCaP) and AR‐negative (PC3 and DU145) metastatic prostate cancer. Strikingly, we observed high synergistic interaction in all five cell lines when CDK2 inhibitor was combined with either Docetaxel or Cisplatin (Fig. 5B). In every cell line, we observed values over 10, which indicates clear synergistic interaction. We confirmed significant combinatorial antiproliferative effects in all five model systems using cell viability assays (Fig. 5C).

**Fig. 6:**
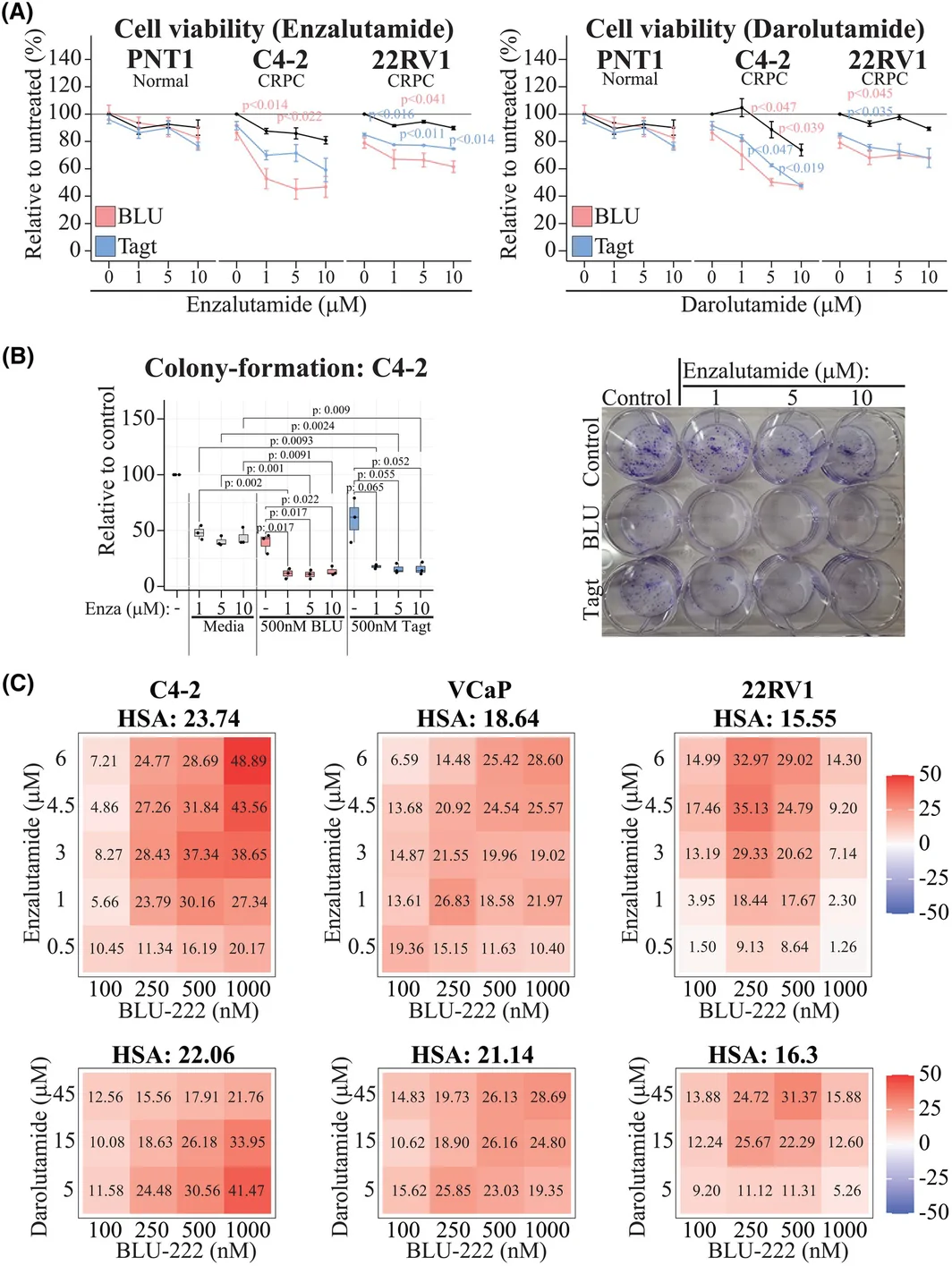
CDK2 inhibition resensitizes castration‐resistant prostate cancer cells to anti‐androgens. (A) CRPC cell lines (C4‐2 and 22RV1) and cell line derived from normal prostate epithelia (PNT1 were treated for 4 days as indicated (BLU‐222: 500 nm and Tagtociclib: 500 nm), viability assay performed and data are the mean of four biological replicates with SEM (each biological replicate comprised of 2 technical replicates for PNT1 and 3 technical replicates for C4‐2 and 22RV1). Paired two‐tailed Student's *t*‐test was used to evaluate statistical significance, which is indicated in comparison between CDK2 inhibitor‐alone and compound of interest‐alone to combination and is color‐coded according to CDK2 inhibitor used. (B) Colony‐formation assay. Cells were treated as indicated and colony‐formation assay performed after 7 days (3 biological replicates with SEM and paired two‐tailed Student's *t*‐test was used to assess the significance; please, refer to figure for the values on significance calculations). (C) Synergy maps of HSA scores between CDK2 inhibitor BLU‐222 and anti‐androgens. Cells were treated for 7 days after which cell viability was assessed using CellTiterGlo. The data are from three biological replicates.

We turned our attention to combinatorial targeting of AR and CDK2. Interestingly, when either of the anti‐androgens, Enzalutamide or Darolutamide, were combined with CDK2 inhibitors, we observed significantly stronger effect on cell viability of the CRPC cell lines (Fig. 6A). The anti‐androgens were less effective to the normal PNT1 cells and did not alter the efficacy of CDK2 inhibition in these cells (Fig. 6A). To acquire further confidence that combined targeting of CDK2 and androgen receptor is more effective on prostate cancer cells, but not cell lines derived from normal prostate tissue, we repeated the combination in LNCaP cells, which represent androgen‐dependent prostate cancer, and RWPE‐1, a cell line representing normal prostate epithelia. Indeed, we detected significant combinatorial effects in LNCaP cells but not in RWPE‐1 cells (Fig. S11A). Colony‐formation assays revealed that combination of either anti‐androgens or complete hormone‐depletion from the media significantly sensitizes both C4‐2 and 22RV1 CRPC cells to CDK2 inhibitor BLU‐222 (Fig. 6B Fig. S11B). We confirmed the importance of high CDK2 levels and activity for CRPC cell response against anti‐androgens using knockdown experiments and colony‐formation assays with both anti‐androgens and complete removal of hormones from the media (Fig. S12). Finally, to validate the combinatorial antiproliferative effects of targeting CDK2 and AR, we performed synergy experiments, which revealed a robust score of over 15 for all AR‐positive cell lines assessed: C4‐2, VCaP, and 22RV1 (Fig. 6C).

In brief, CDK2 inhibition sensitizes prostate cancer cells to both cytotoxic compounds and anti‐androgens. It is important to remember that despite originating from noncancer tissue, both PNT1 and RWPE‐1 cell lines have been immortalized, and further validation of both the efficacy and safety of CDK2 inhibitor‐based therapy in more complex model systems would be beneficial. Inhibitors of AR are widely used and offer an attractive avenue for further development of CDK2 inhibitors to eventual clinical use.

### 3.5 CDK2 inhibition stimulates androgen receptor activity

**Fig. 7:**
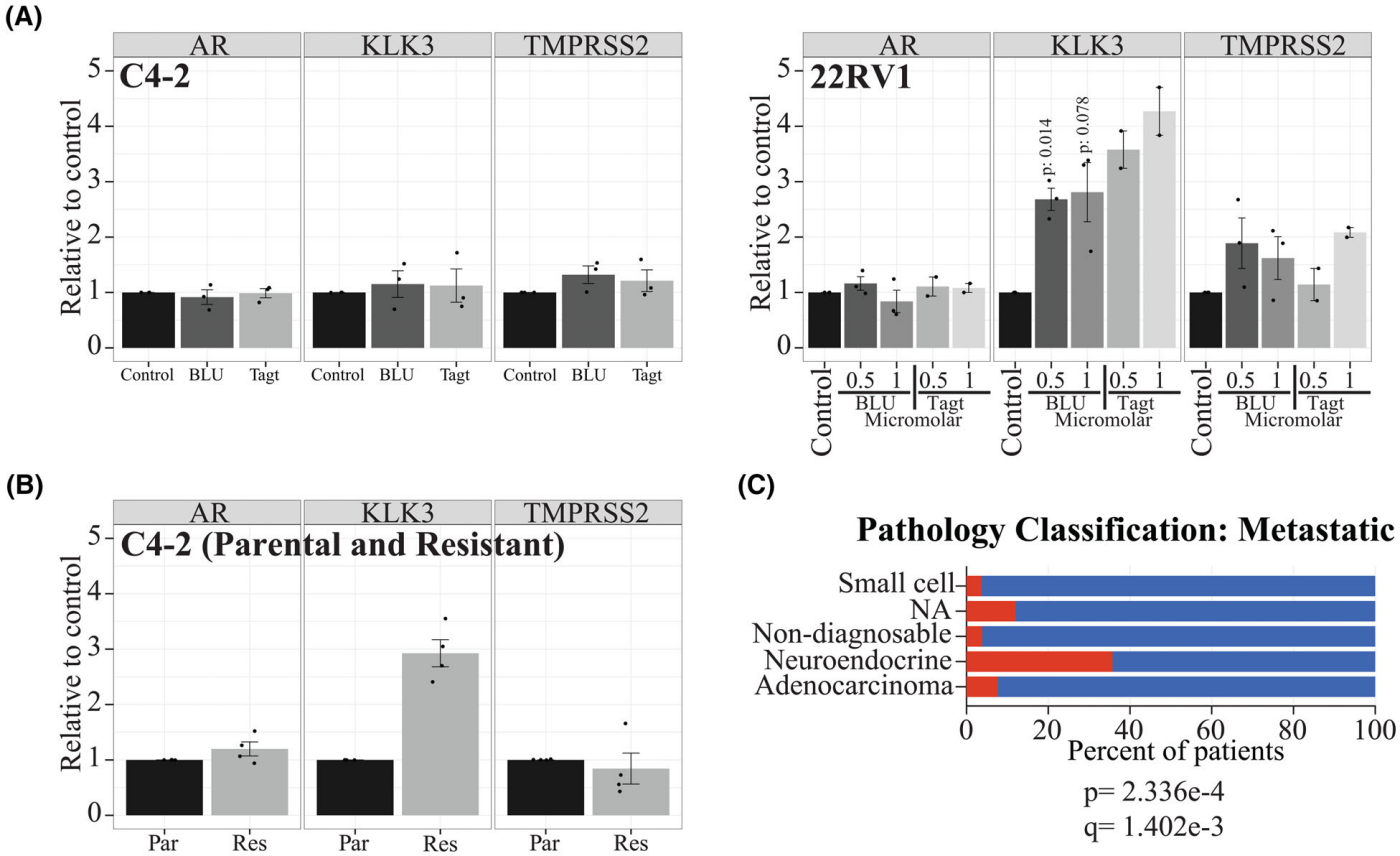
Adaptation to CDK2 inhibition stimulates androgen receptor (AR) activity acutely and chronically. (A) CRPC cell lines C4‐2 and 22RV1 were treated with BLU‐222 or Tagtociclib for 48 h. mRNA levels of AR and AR target genes were measured by qPCR. Boxplots represent data from 3 biological replicates for all genes in C4‐2 and 22RV1, except for 22RV1‐samples treated with 0.5 μm and 1 μm Tagtociclib, for which two biological replicates were analyzed. The expression of the genes of interest is presented relative to actin. Error bars represent the SEM. (B) mRNA levels of AR and AR target genes, KLK3 (Kallikrein Related Peptidase 3), and TMPRSS2 (transmembrane Serine Protease 2) were quantified by qPCR in parental and BLU‐222‐resistant C4‐2 cells. Data are from four biological replicates with SEM. (C) Pathology classification of prostate tumors over‐expressing *CDK2* and/or *CCNE1* and/or *CCNE2* (exp > 2) accessed through the cBioPortal in the dataset “Metastatic Prostate Adenocarcinoma (SU2C/PCF Dream Team, PNAS 2019)”. A total of 429 patient samples were analyzed, and statistical comparisons were performed using the Wilcoxon test, and multiple‐hypothesis testing correction was performed using the Benjamini–Hochberg false‐discovery‐rate method.

Next, we wanted to understand if CDK2 inhibition alters AR levels and/or activity acutely and/or chronically. Interestingly, CDK2 inhibition stimulated AR activity in both of the CRPC models assessed (C4‐2 and 22RV1) as determined by the expression of AR target genes *KLK3* and *TMPRSS2*, which were increased up to fourfold, but the levels of AR remained unchanged at both mRNA‐ and protein levels (Fig. 7A and Fig. S13). We observed a similar response in our CDK2 inhibitor‐resistant cell line revealing a threefold increase in KLK3 (Fig. 7B). Evidently, only a subset of AR target genes is affected and altered expression of AR does not explain this. It is important to remember that combined inhibition of CDK2 and AR induces synergistic antiproliferative effects (Fig. 6C). These data propose that AR may gain new targets, and/or the activity of AR coregulators is altered. Indeed, it is well‐established that AR cistrome is significantly rewired during prostate epithelia transformation and in response to therapy [42, 43, 44]. However, establishing how CDK2 inhibition alters AR‐cistrome goes beyond the scope of this manuscript, where we probe if targeting CDK2 has potential as prostate cancer therapy.

We wanted to understand if altered CDK2 activity is associated with altered AR signaling also in patient tumors and used the metastatic prostate cancer sample set available through the cBioPortal to assess this. Interestingly, increased expression of CDK2 and/or its cyclins was significantly associated with the neuroendocrine‐phenotype (Fig. 7C). These data show that CDK2 inhibition increases androgen receptor activity in CRPC cells, which can be therapeutically targeted using anti‐androgens.

In brief, here we show that development of prostate cancer is associated with increased dependency on CDK2, which becomes an actionable point of vulnerability as cells develop the CRPC phenotype. We anticipate that appropriate sequencing of either targeted therapies or cytotoxic compounds is required for efficacy in future clinical trials with CDK2 inhibitors against prostate cancer.

## 4 Discussion

Dysregulation of the cell cycle enables cancer cells to proliferate rapidly but may also open therapeutic vulnerabilities, which has prompted the development of targeted therapies. Here, we show that the development of CRPC leads to an acquired dependency on CDK2 while the role of CDKs4/6 diminishes in parallel. Using two CDK2 inhibitors in clinical development along with knockdown strategies, we reveal that CDK2 forms a combinatorial antiproliferative target with the existing prostate cancer therapeutics, most notably radiotherapy, Docetaxel, Cisplatin, and anti‐androgens.

The cyclic regulation of specific cyclins drives the initiation, execution, and termination of the cell cycle, and cancer cells appear to be more dependent on signaling through specific cyclin‐CDK pairs. Here, we show that the development of CRPC is associated with an increased expression of mitotic cyclins, which likely represents a higher proportion of actively cycling cells within the tumor rather than overall increased expression in only a few cells (Fig. S2). In fact, there are clinically utilized tests that measure the expression of cell cycle mRNAs and predict how aggressive the patient's tumor is, such as the Prolaris Score [45]. Strikingly, however, we show here that the CRPC cells downregulate CCNDs and upregulate CCNEs, implying an acquired dependency on CDK2 (Fig. 1B).

We show that CRPC cells are dependent on CDK2, which we propose is explained by the underlying factor driving the cell cycle progression. We show that hormone‐dependent, CRPC, and AR‐negative prostate cancer cells are sensitive to CDK2 inhibitors BLU‐222 and Tagtociclib (Fig. 2A,B,E). Earlier, despite the initial positive results using CDK4/6 inhibitors as a prostate cancer therapy, these compounds have been discontinued due to lack of efficacy [16, 46]. This is likely explained by the fact that in prostate cancer multiple growth stimuli synergize to promote cell cycle, in particular androgens and epidermal growth factor, which activate CDK2 but not CDK4 or CDK6 [47]. Androgen receptor drives the expression of a large number of cell cycle regulators but, curiously, does not prominently affect the expression of CDK4/6 or their cyclins [48]. Instead, it acts by downregulating the endogenous inhibitor of both CDK2 and CDK4, p27 [47, 49]. Accordingly, over‐expression of G1/S cyclins is not sufficient to confer androgen‐independent proliferation [50]. In a striking contrast to this, the hormone‐positive breast cancer cells drive the expression of the *CCND* gene through a tissue‐specific enhancer [51]. Accordingly, these cells are particularly dependent on high CDK4/6 activity, as initially demonstrated through *in vitro* and animal experiments [12, 27], and later on in clinical trials and eventual approval as therapy [52, 53]. However, the emergence of resistance remains an issue in the case of CDK4/6 inhibitors [52], as is likely the case for CDK2 inhibitors based on the data presented in this manuscript (Fig. 3). It is curious that breast cancer cells maintain a tissue‐specific enhancer to sustain *CCND* gene expression [51]. When the activity of cell cycle CDKs is impaired, this appears to increase lineage plasticity and therapy resistance, which may, in fact, be caused due to compounds targeting these CDKs. Development of highly potent anti‐androgens has driven prostate cancer cells to develop therapy resistance through increased lineage plasticity, and this remains a serious issue in the field [54, 55, 56]. Increased lineage plasticity could occur, for example, because the epigenetic marks are installed during the cell cycle to the newly synthesized strand [57], and impairing this process could lead to incomplete or excessive marks. In fact, cell cycle CDKs phosphorylate the catalytic subunit of Polycomb repressive complex 2, EZH2, which is important for targeting EZH2 to correct loci [58]. Importantly, however, we identify rational strategies to circumvent the emerging resistance and augment the efficacy of CDK2 inhibitor‐based therapy. It is tempting to propose that inhibition of cell cycle kinase(s) together with more cancer cell‐targeted therapy could suppress increased lineage plasticity and emergence of therapy resistance.

We show that CDK2 inhibition triggers transcription‐replication conflicts in prostate cancer cells, which opens a combinatorial treatment strategy. Using immunofluorescence, we demonstrated accumulation of R‐loops, particularly in the densely packed regions of the nucleus, likely representing nucleoli where ribosomal RNA is synthesized (Fig. 4A). This prompted us to ask if CDK2 inhibitors sensitize cells to compounds interfering directly with ribosomal RNA synthesis through RNA Pol I inhibitors or translation by targeting mTOR; however, while the effects were significant, they were rather modest (Fig. 4B). Nevertheless, we noted robust accumulation of R‐loops, which are known to activate the innate immune response [59, 60, 61]. In the future, it is therefore of high interest to establish if CDK2 inhibitors can stimulate antitumor immunity, as this could lead to curative therapy. In support of this, we show that radiotherapy enhances the antiproliferative effect of CDK2 inhibition (Fig. 4C and Fig. S9).

We developed a CDK2 inhibitor resistant cell line and evaluated how these cells escape from the initially cell cycle‐halting therapy (Figs 3, 5 and 6). The experience from treating breast cancer patients with CDK4/6 inhibitors has revealed escape mechanisms particularly through CDK2 and RB1, which provide rational targeted therapy with the recently developed CDK2 inhibitors [30, 62]. In support of this, we show that resistance to CDK2 inhibition can result in increased dependency on cell cycle kinases CDK4/6 (Fig. 3E). However, current clinical trials assessing the CDK2 and CDK4/6 inhibition combinations are either terminated with no data disclosed (NCT05252416) or are not actively recruiting (NCT04553133). In addition, the combination of CDK2 and CDK4/6 inhibitors, while likely effective against cancer cells, can be expected to result in on‐target toxicity due to effects on normal cells. Therefore, we set up a library of therapies currently used against prostate cancer, which revealed a synergistic interaction between CDK2 inhibition and anti‐androgens in multiple CRPC cell lines (Fig. 6C). Interestingly, CDK2 inhibition also stimulated AR activity (Fig. 7A,B). These data suggest that inhibiting CDK2 makes CRPC cells more reliant on high AR activity. Curiously, this is the same principal rationale as utilized against breast cancer where the anti‐estrogen receptor therapy is successfully combined with CDK4/6 inhibitors [63].

It is possible that remodeling the cell cycle program through altered activity of cell cycle kinases contributes to acquisition of distinct cellular lineages that confer growth advantage. This can occur through CDK2‐mediated phosphorylation of EZH2 in prostate cancer cells exposed to potent anti‐androgens, albeit other kinases may also phosphorylate EZH2 [58, 64, 65]. Our data provide the first evidence that high CDK2 activity is important for neuroendocrine prostate cancer cells (Fig. 7C); however, the role of CDK2 in neuroendocrine prostate cancer clearly merits a follow‐up study.

In summary, CDK2 inhibitors provide an interesting avenue to target prostate cancer. Based on the existing clinical trials, it is possible that some prostate cancer patients are treated with CDK2 inhibitors as part of those (e.g., NCT05262400 and NCT05252416). In the future, it would be useful to establish the efficacy of the potential combination therapies discovered in this study using either animal models or patient‐derived organoids. In brief, both the existing literature and the data presented in this manuscript underscore the importance of the underlying tissue‐specific transcriptional program as a targetable vulnerability in combination with CDK inhibition.

## 5 Conclusions

In brief, we show that prostate cancer cells upregulate E‐type cyclins (CCNE1/2) and are sensitive to CDK2 inhibitors. Our data provide rationale for further development of a CDK2‐based intervention strategy in combination with existing prostate cancer therapies.

## Conflict of interest

The authors declare no conflict of interest.

## Author contributions

JC evaluated the expression of factors of interest in patient samples, performed the combinatorial screen with prostate cancer‐relevant therapies, generated all figures in R and wrote the initial draft of the introduction, discussion, and figure legends. AM performed a significant part of the wet lab experiments. SY assisted AM in experiments and contributed to manuscript writing. HMI conceptualized the study, acquired resources and wrote the initial draft of the manuscript. All authors provided critical feedback on the manuscript.

## Supporting information


**Fig. S1.** Primers used in this study.
**Fig. S2.** Mitotic cyclins and cyclin‐dependent kinase 1 (CDK1) are over expressed in primary and metastatic prostate cancer. mRNA expression levels of cyclins and cyclin‐dependent kinases were analyzed across normal prostate tissue, primary prostate cancer, metastatic prostate cancer, and castration‐resistant prostate cancer using patient data obtained from (reference 1 in Supporting Information) accessed through betastasis.com. A total of 177 samples were analyzed, including 28 normal prostate samples, 130 primary prostate cancer samples, 8 metastasized non‐castrate prostate cancer samples and 11 metastasized castrate prostate cancer samples. Error bars represent the standard error of the mean (SEM) and unpaired Mann–Whitney‐Wilcoxon test was used to evaluate statistical significance. Please, refer to figure for the values on significance‐calculations.
**Fig. S3.** CDK2 inhibition decreases prostate cancer spheroid growth. C4‐2 and 22RV1 cells were plated in MatriGel 1 day prior to treatments. After 1 week, example images were taken and the spheroids lysed with CellTiterGlo‐reagent. Data shown is the mean of three biological replicates with SEM. Paired two‐tailed Student's *t*‐test was used to evaluate statistical significance. Please, refer to figure for the values on significance‐calculations.
**Fig. S4.** Example images of colony‐formation experiment. Colony‐formation assay after 7 days treatment with BLU‐222 and Tagtociclib (4 biological replicates).
**Fig. S5.** Knockdown of CDK2 significantly decreases colony‐formation. Colony formation assay after 7 days of CDK2 knockdown in C4‐2 cell line (3 biological replicates, one sample *t*‐test was used to evaluate statistical significance and error bars represent the SEM). Please, refer to the figure for the values on significance‐calculations. Western blot was used to confirm successful depletion of CDK2 after the knockdown (representative of three biological replicates).
**Fig. S6.** Expression of selected cyclin‐mRNAs. RT‐qPCR analysis of parental and BLU‐222 resistant C4‐2 cells after the indicated treatments. Data are from 3 biological replicates for the parental C4‐2 cells treated for 24 and 72 h, and 4 biological replicates for the comparison between parental and BLU‐222‐resistant C4‐2 cells, error bars represent the SEM and statistical significance is evaluated by one sample *t*‐test and is indicated for the samples that reach significance. Please, refer to figure for the values on significance‐calculations.
**Fig. S7.** Expression of selected CDK‐mRNAs. RT‐qPCR analysis of parental and BLU‐222 resistant C4‐2 cells after the indicated treatments. Data are from 3 biological replicates for the parental C4‐2 cells treated for 24 and 72 h, and 4 biological replicates for the comparison between parental and BLU‐222‐resistant C4‐2 cells, error bars represent the SEM, statistical significance is evaluated by one sample *t*‐test and is indicated for the samples that reach significance. Please, refer to figure for the values on significance‐calculations.
**Fig. S8.** Validation of S9.6 immunofluorescence and effect of depleting CDK2 activity on proteins of interest. (A) C4‐2 cells were treated with BLU‐222 (500 nm) for 3 days and R‐loop accumulation was detected using immunofluorescence (the S9.6 antibody). Prior to antibody staining, samples were treated with RNase H, as indicated. Scale bar is in the lower right corner in the images and indicates length of 10 micrometers. (B) C4‐2 cells were treated as indicated for 3 days and western blot was used to detect proteins of interest. (C) C4‐2 cells were transfected with CDK2 targeting siRNAs for 3 days, and protein levels of CDK2, p53 and actin were analyzed by western blotting. Data is representative of three biological replicates.
**Fig. S9.** Example images of colony‐formation experiment (C4‐2 and 22RV1) and colony‐formation assay for 22RV1. (A) Example images. (B) Colony‐formation assay. Cells were either not radiated (C), first radiated and treated after 1 day (R > C), first treated and then radiated after 1 day (C > R) or radiated and treated at the same time (CR). After 7 days, the colonies were detected. Data is from 4 biological replicates with SEM. Paired two‐tailed Student's *t*‐test was used to evaluate statistical significance and the value reported is in comparison to both radiation‐alone and CDK2 inhibitor‐alone (whichever is higher, is reported). Please, refer to figure for the values on significance‐calculations.
**Fig. S10.** Targeted compound screen and subsequent validation reveal antiproliferative effects between CDK2 inhibition and Cabazitaxel or Carboplatin. Castration resistant prostate cancer (CRPC) cell lines (C4‐2 and 22RV1) and cell line derived from normal prostate epithelia (PNT1) were treated for 4 days as indicated (BLU‐222: 500 nM and Tagtociclib: 500 nm), viability assay performed and data is the mean of four biological replicates with SEM (each biological replicate comprised 2 technical replicates for PNT1 and 3 technical replicates for C4‐2 and 22RV1). Paired two‐tailed Student's *t*‐test was used to evaluate statistical significance, which is indicated in comparison between CDK2 inhibitor‐alone and compound of interest‐alone to combination and is color‐coded according to CDK2 inhibitor used.
**Fig. S11.** Combined inhibition of CDK2 and androgen receptor induces anti proliferative effects on prostate cancer cells. (A) Prostate cancer cells (LNCaP) and cell line derived from normal prostate epithelia (RWPE‐1) were treated for 4 days as indicated (BLU‐222: 500 nm and Tagtociclib: 500 nm), viability assay performed and data is the mean of four biological replicates (each biological replicate comprised 2 technical replicates for LNCaP and 3 technical replicates for RWPE‐1) with SEM. Paired two‐tailed Student's *t*‐test was used to evaluate statistical significance, which is indicated in comparison between CDK2 inhibitor‐alone and compound of interest‐alone to combination and is color‐coded according to CDK2 inhibitor used. (B) Colony‐formation assay. Cells were treated as indicated and colony‐formation assay performed after 7 days (3 biological replicates with SEM and paired two‐tailed Student's *t*‐test was used to assess the significance). Please, refer to figure for the values on significance‐calculations.
**Fig. S12.** Knockdown of CDK2 sensitizes castration‐resistant prostate cancer cells to anti‐androgens. CDK2 was knocked down for a day after which C4‐2 cells were treated as indicated and colony‐formation assay performed after 7 days (3 biological replicates with SEM and paired samples two‐tailed Student's *t*‐test was used to assess the significance). Please, refer to figure for the values on significance‐calculations. Darolutamide and Enzalutamide dose: 5 μm, CSS = androgen starvation (charcoal‐stripped serum).
**Fig. S13.** CDK2 inhibition does not alter AR protein levels after 24 h treatment. C4‐2 cells were treated as indicated for 24 h and samples analyzed using western blot. Please, note that these are the same samples as those in main Fig. 2C and actin western blot is the same as in main Fig. 2C. Data is representative of two biological replicates.

## Data Availability

All the data and materials are presented as part of this manuscript and/or the S1.
