# Supplementary material for: Castration‐resistant prostate cancer cells are addicted to the high activity of cyclin‐dependent kinase 2
Source: Mol Oncol. 2026 Sep 2:10.1002/1878-0261.70319. Online ahead of print. doi: 10.1002/1878-0261.70319 (PMC13536838; doi:10.1002/1878-0261.70319)
Supplement: Supplementary file 1 [file MOL2-9999-0-s001.pdf]

Supplementary figures, additional information and supplementary figure legends

Additional information on patient samples accessed using online tools

For **Fig. 1B**, the analysis was performed using the Taylor et al. 2010 prostate cancer dataset, "Integrative Genomic Profiling of Human Prostate Cancer (1)," accessed through Betastasis. In that dataset, a total of 177 samples were analyzed, including 28 normal prostate samples, 130 primary prostate cancer samples, 8 metastasized non-castrate prostate cancer samples and 11 metastasized castrate prostate cancer samples. We used the processed expression values available through Betastasis (https://www.betastasis.com/). For each gene, expression values were normalized to the mean expression of the normal prostate-group, such that the average normal expression was set to 1. Each disease state was compared with the reference value of 1 using unpaired Mann-Whitney-Wilcoxon test to evaluate statistical significance. The analysis was performed using the processed expression values available through Betastasis; therefore, no additional batch correction was applied in our downstream analysis. Samples were analyzed as independent patient-level specimens based on the available dataset annotation.

For **Fig. 1C**, the analysis was performed using the Prostate Adenocarcinoma TCGA Firehose Legacy cohort accessed through cBioPortal. A total of 491 samples with available CDK2 alteration and clinical annotation data were analyzed. The data was presented directly as obtained from cBioPortal. Clinical variables were compared between CDK2-altered and CDK2-unaltered groups using the Wilcoxon test implemented in the cBioPortal. cBioPortal-reported q-values were used to account for multiple testing using the Benjamini–Hochberg false-discovery-rate method.

For **Fig. 7C**, the analysis was performed using the Metastatic Prostate Adenocarcinoma SU2C/PCF Dream Team PNAS 2019 cohort (2) accessed through cBioPortal. A total of 429 patient samples were analyzed. The data is presented directly as obtained from cBioPortal, without additional downstream normalization or batch correction. Statistical comparisons were performed using the Wilcoxon test, and multiple-hypothesis testing correction was performed using the Benjamini–Hochberg false-discovery-rate method.

Primer	Sequence
CCNE1_F	AAGGAGCGGGACACCATGA
CCNE1_R	ACGGTCACGTTTGCCTTCC
CCNE2_F	TCAAGACGAAGTAGCCGTTTAC
CCNE2_R	TGACATCCTGGGTAGTTTTCCTC
CCND1_F	GCTGCGAAGTGGAAACCATC
CCND1_R	CCTCCTTCTGCACACATTTGAA
CCND2_F	ACCTTCCGCAGTGCTCCTA
CCND2_R	CCCAGCCAAGAAACGGTCC
CCND3_F	TACCCGCCATCCATGATCG
CCND3_R	AGGCAGTCCACTTCAGTGC
CCNB1_F	AATAAGGCGAAGATCAACATGGC
CCNB1_R	TTTGTTACCAATGTCCCCAAGAG
CCNB2_F	CCGACGGTGTCCAGTGATTT
CCNB2_R	TGTTGTTTTGGTGGGTTGAACT
CCNB3_F	ATGAAGGCAGTATGCAAGAAGG
CCNB3_R	CATCCACACGAGGTGAGTTGT
CCNA1_F	GAGGTCCCGATGCTTGTCAG
CCNA1_R	GTTAGCAGCCCTAGCACTGTC
CCNA2_F	CGCTGGCGGTACTGAAGTC
CCNA2_R	GAGGAACGGTGACATGCTCAT
CDK1_F	AAACTACAGGTCAAGTGGTAGCC
CDK1_R	TCCTGCATAAGCACATCCTGA
CDK2_F	CCAGGAGTTACTTCTATGCCTGA
CDK2_R	TTCATCCAGGGGAGGTACAAC
CDK4_F	ATGGCTACCTCTCGATATGAGC
CDK4_R	CATTGGGGACTCTCACACTCT
CDK6_F	GCTGACCAGCAGTACGAATG
CDK6_R	GCACACATCAAACAACCTGACC
AR_F	TCGACCATTTCTGACAACGC
AR_R	GAAGCTGTTCCCCTGGACTC
KLK3_F	GCAGCATTGAACCAGAGGAG
KLK3_R	AGAACTGGGGAGGCTTGAGT
TMPRSS2_F	GTCCCCACTGTCTACGAGGT
TMPRSS2_R	CAGACGACGGGGTTGGAAG
ACTIN_For	TGGGACGACATGGAGAAAAT
ACTIN_Rev	AGAGGCGTACAGGGATAGCA

Supplementary figure 1. Primers used in this study.

A

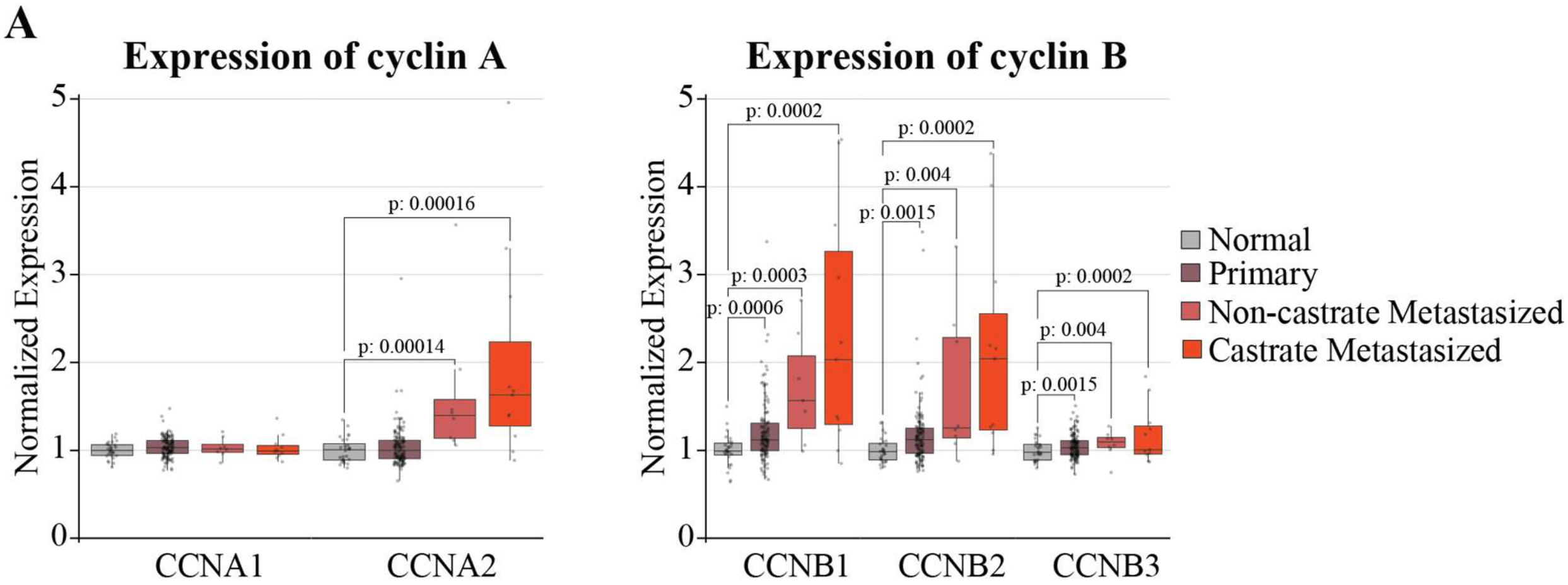

B

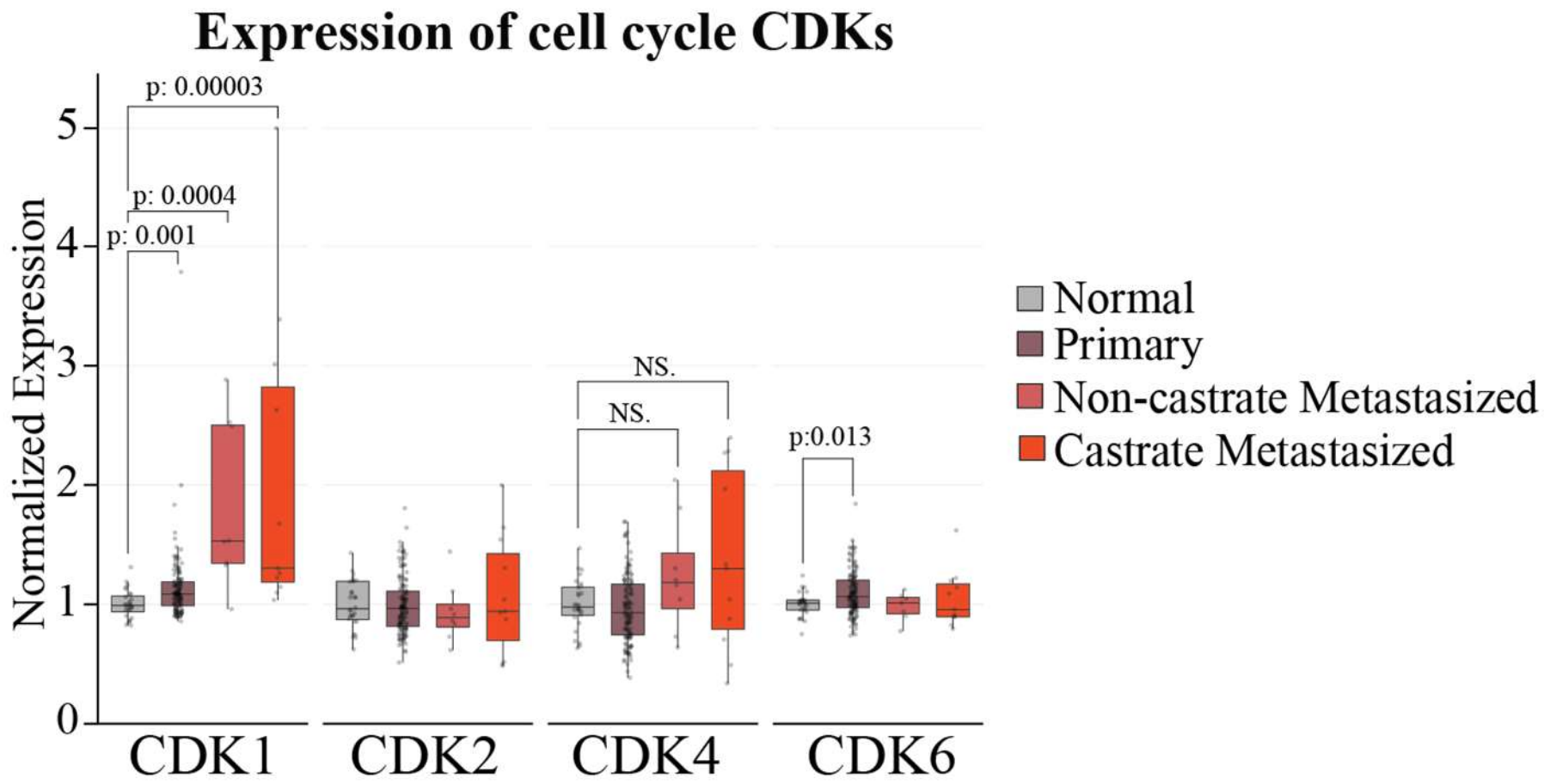

Supplementary figure 2. Mitotic cyclins and cyclin-dependent kinase 1 (CDK1) are over-expressed in primary and metastatic prostate cancer. mRNA expression levels of cyclins and cyclin-dependent kinases were analyzed across normal prostate tissue, primary prostate cancer, metastatic prostate cancer, and castration-resistant prostate cancer using patient data obtained from (reference 1 in Supporting Information) accessed through betastasis.com. A total of 177 samples were analyzed, including 28 normal prostate samples, 130 primary prostate cancer samples, 8 metastasized non-castrate prostate cancer samples and 11 metastasized castrate prostate cancer samples. Error bars represent the standard error of the mean (SEM) and unpaired Mann-Whitney-Wilcoxon test was used to evaluate statistical significance. Please, refer to figure for the values on significance-calculations.

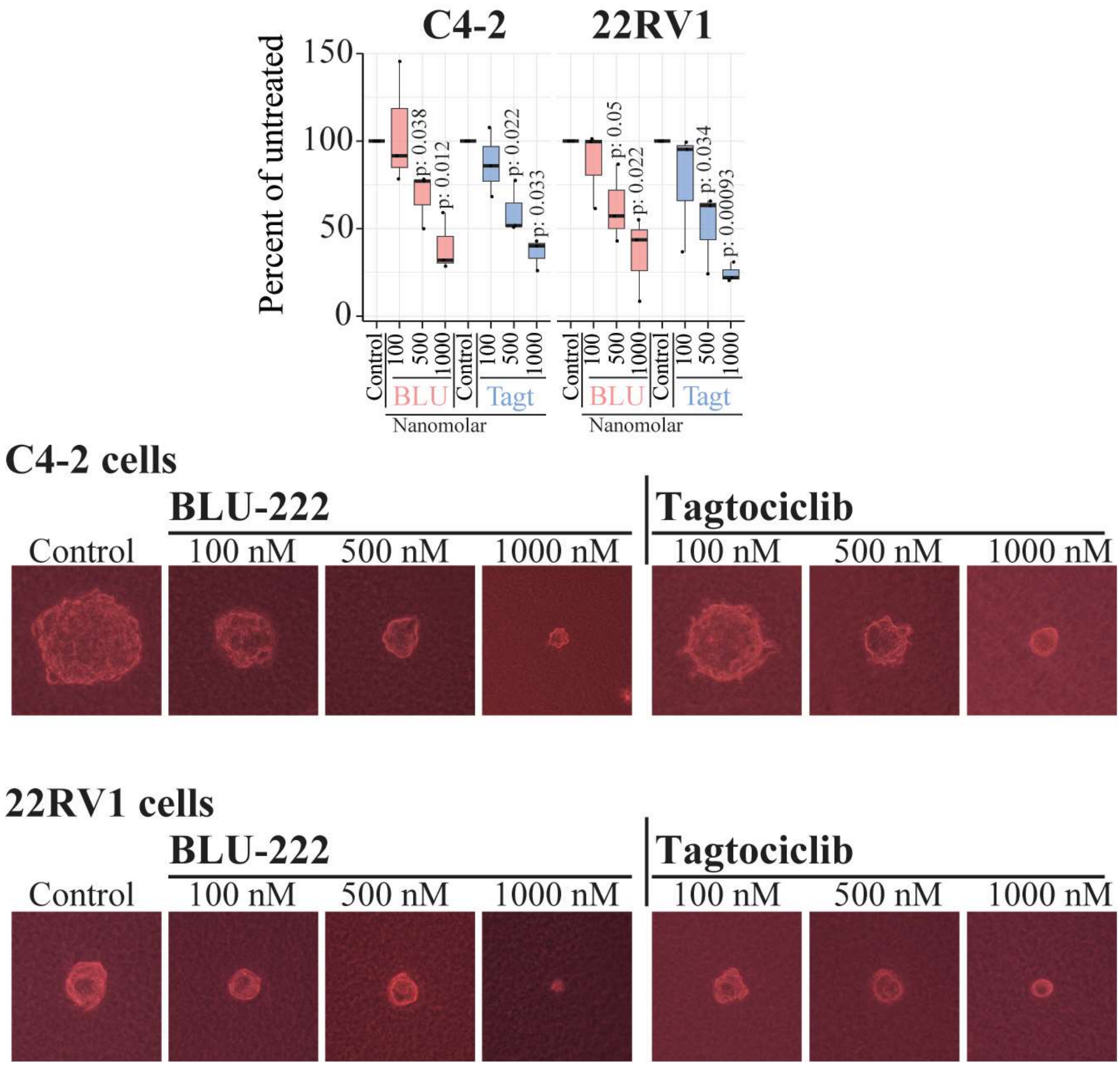

Supplementary figure 3. CDK2 inhibition decreases prostate cancer spheroid growth. C4-2 and 22RV1 cells were plated in MatriGel one day prior to treatments. After one week, example images were taken and the spheroids lysed with CellTiterGlo-reagent. Data shown is the mean of three biological replicates with SEM. Paired two-tailed Student's *t*-test was used to evaluate statistical significance. Please, refer to figure for the values on significance-calculations.

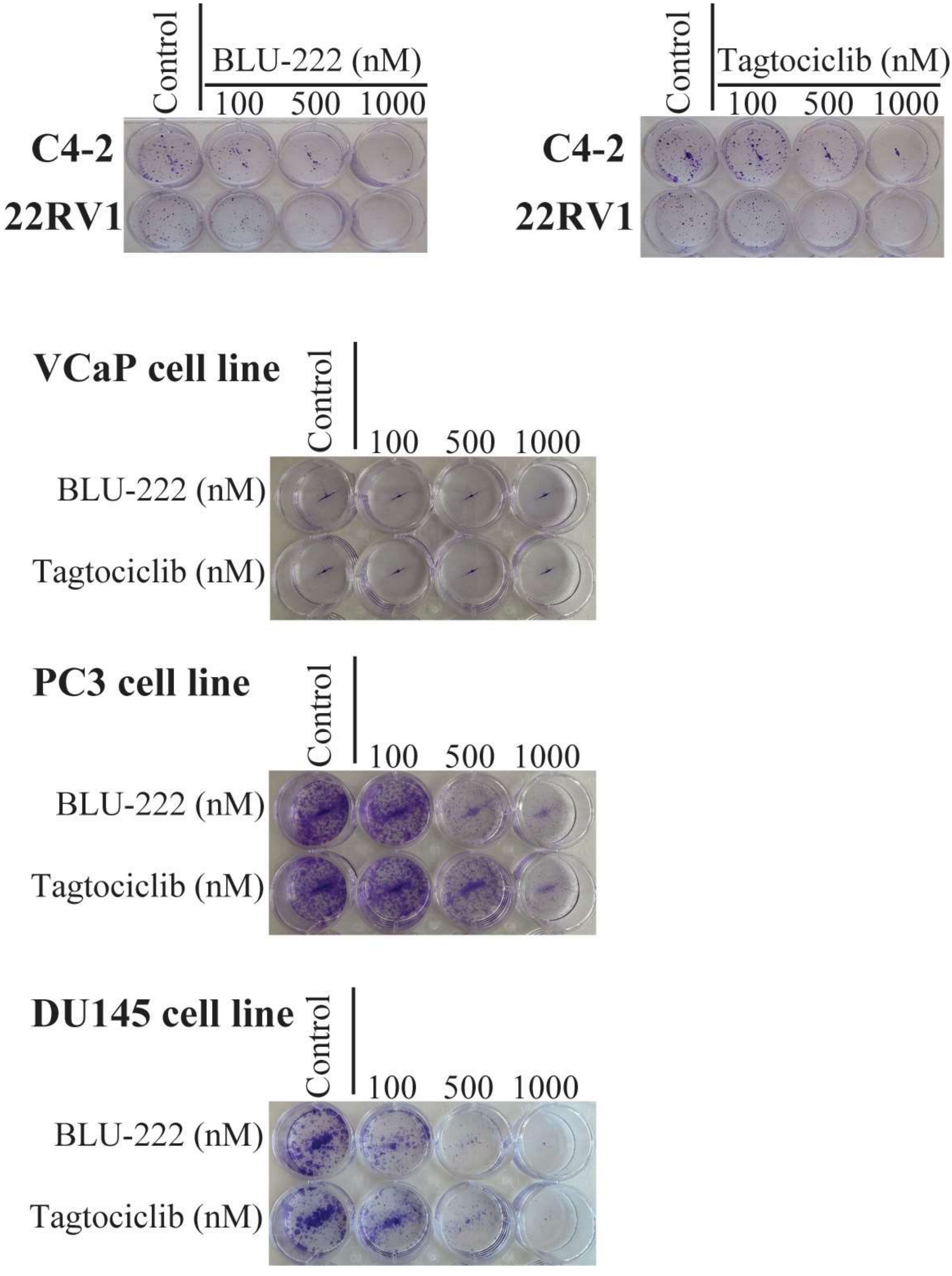

Supplementary figure 4. Example images of colony-formation experiment. Colony-formation assay after 7 days treatment with BLU-222 and Tagtociclib (4 biological replicates).

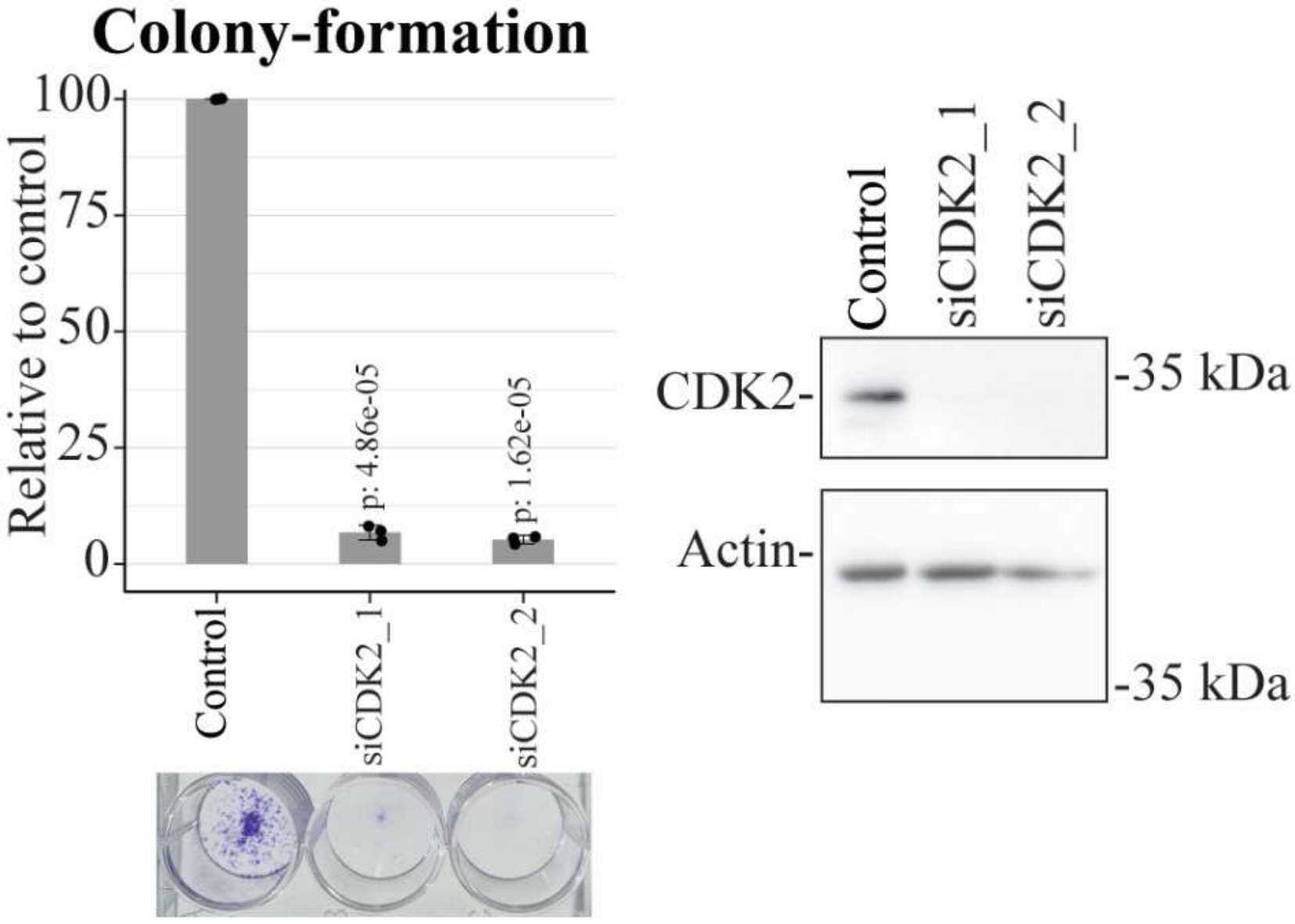

Supplementary figure 5. Knockdown of CDK2 significantly decreases colony-formation. Colony formation assay after 7 days of CDK2 knockdown in C4-2 cell line (3 biological replicates, one-sample *t*-test was used to evaluate statistical significance and error bars represent the SEM. Please, refer to the figure for the values on significance-calculations. Western blot was used to confirm successful depletion of CDK2 after the knockdown (representative of three biological replicates).

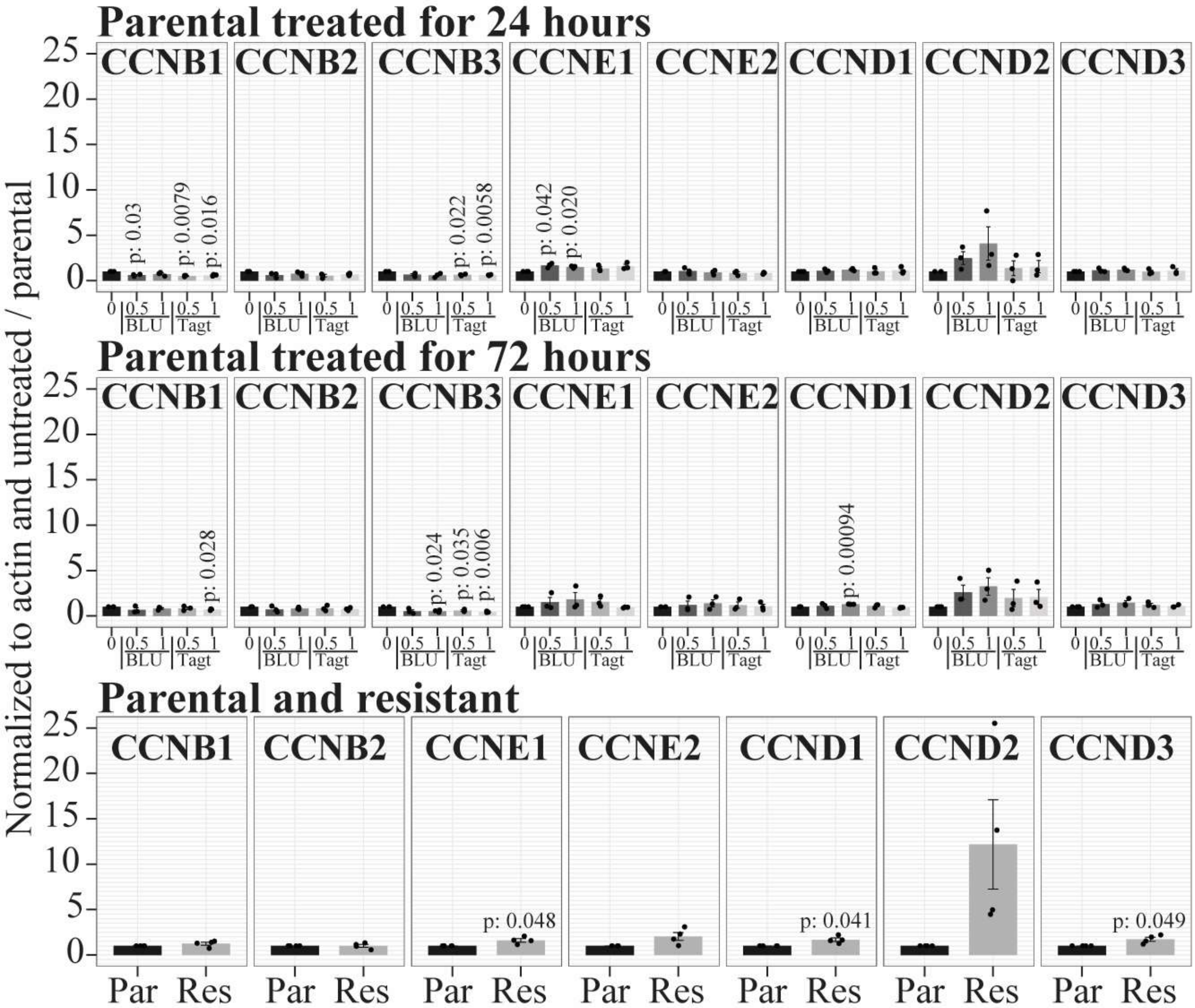

Supplementary figure 6. Expression of selected cyclin-mRNAs. RT-qPCR analysis of parental and BLU-222 resistant C4-2 cells after the indicated treatments. Data are from 3 biological replicates for the parental C4-2 cells treated for 24 and 72 hours, and 4 biological replicates for the comparison between parental and BLU-222-resistant C4-2 cells, error bars represent the SEM and statistical significance is evaluated by one sample *t*-test and is indicated for the samples that reach significance. Please, refer to figure for the values on significance-calculations.

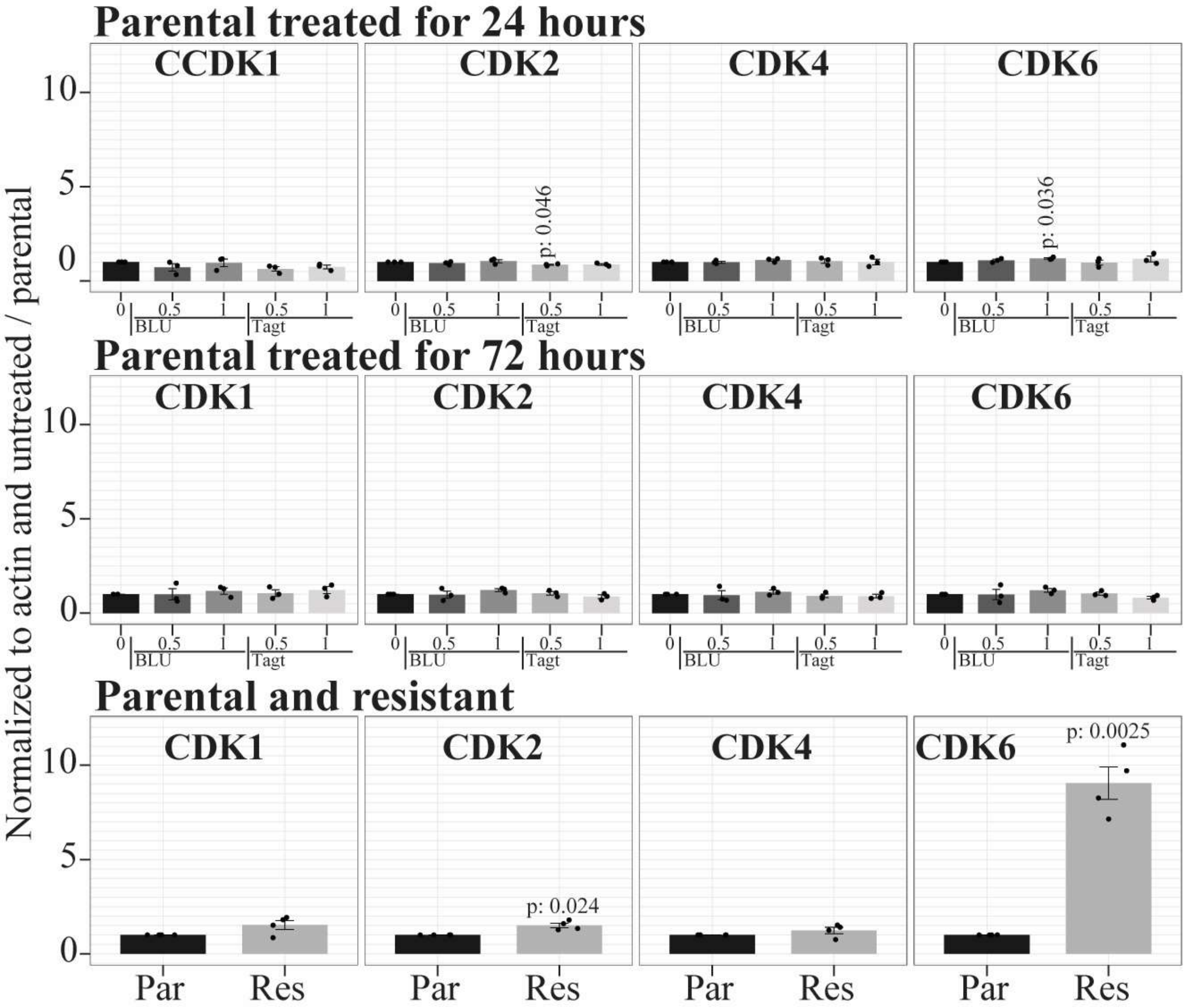

Supplementary figure 7. Expression of selected CDK-mRNAs. RT-qPCR analysis of parental and BLU-222 resistant C4-2 cells after the indicated treatments. Data are from 3 biological replicates for the parental C4-2 cells treated for 24 and 72 hours, and 4 biological replicates for the comparison between parental and BLU-222-resistant C4-2 cells, error bars represent the SEM, statistical significance is evaluated by one sample *t*-test and is indicated for the samples that reach significance. Please, refer to figure for the values on significance-calculations.

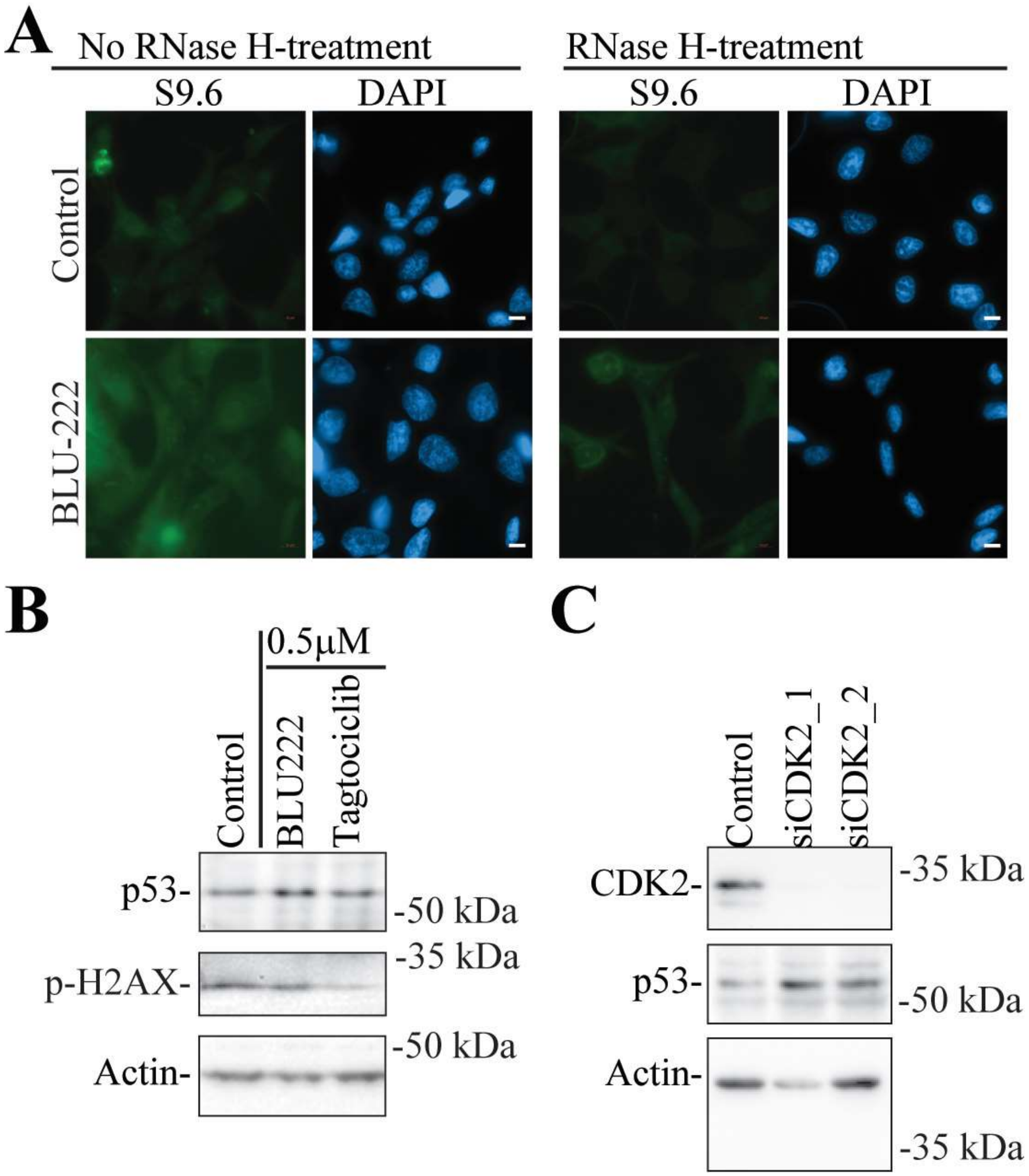

Supplementary figure 8. Validation of S9.6 immunofluorescence and effect of depleting CDK2 activity on proteins of interest. A) C4-2 cells were treated with BLU-222 (500 nM) for 3 days and R-loop accumulation was detected using immunofluorescence (the S9.6 antibody). Prior to antibody-staining, samples were treated with RNase H, as indicated. Scale bar is in the lower right corner in the images and indicates length of 10 micrometers. **B)** C4-2 cells were treated as indicated for 3 days and western blot was used to detect proteins of interest. **C)** C4-2 cells were transfected with CDK2-targeting siRNAs for 3 days, and protein levels of CDK2, p53 and actin were analyzed by western blotting. Data is representative of three biological replicates.

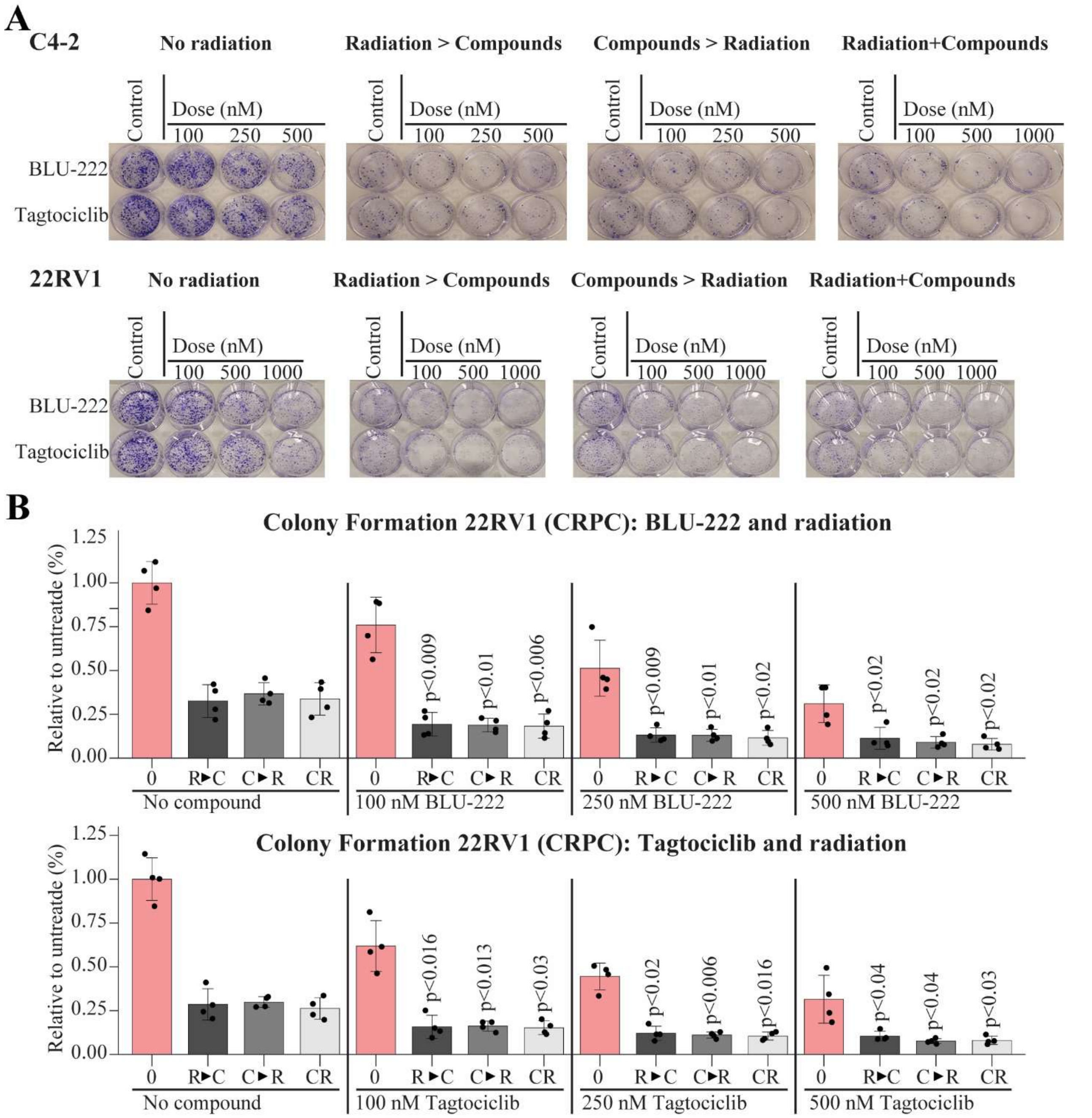

Supplementary figure 9. Example images of colony-formation experiment (C4-2 and 22RV1) and colony-formation assay for 22RV1. A) Example images. **B)** Colony-formation assay. Cells were either not radiated (C), first radiated and treated after 1 day (R>C), first treated and then radiated after 1 day (C>R) or radiated and treated at the same time (CR). After 7 days, the colonies were detected. Data is from 4 biological replicates with SEM. Paired two-tailed Student's t-test was used to evaluate statistical significance and the value reported is in comparison to both radiation-alone and CDK2 inhibitor-alone (whichever is higher, is reported). Please, refer to figure for the values on significance-calculations.

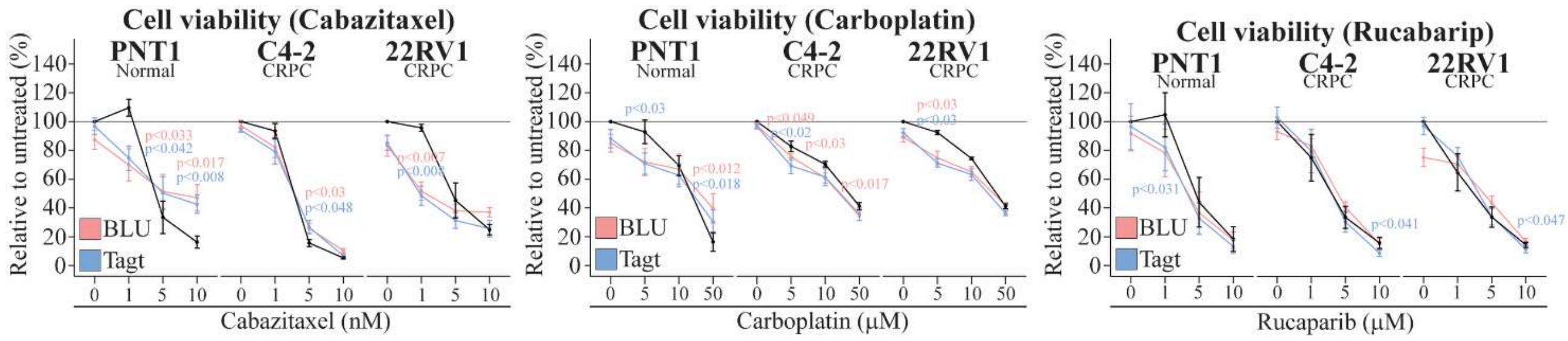

Supplementary figure 10. Targeted compound screen and subsequent validation reveal anti proliferative effects between CDK2 inhibition and Capazitaxel or Carboplatin. Castration resistant prostate cancer (CRPC) cell lines (C4-2 and 22RV1) and cell line derived from normal prostate epithelia (PNT1) were treated for 4 days as indicated (BLU-222: 500 nM and Tagtociclib: 500 nM), viability assay performed and data is the mean of four biological replicates with SEM (each biological replicate comprised 2 technical replicates for PNT1 and 3 technical replicates for C4-2 and 22RV1). Paired two-tailed Student's t-test was used to evaluate statistical significance, which is indicated in comparison between CDK2 inhibitor-alone and compound of interest-alone to combination and is color-coded according to CDK2 inhibitor used.

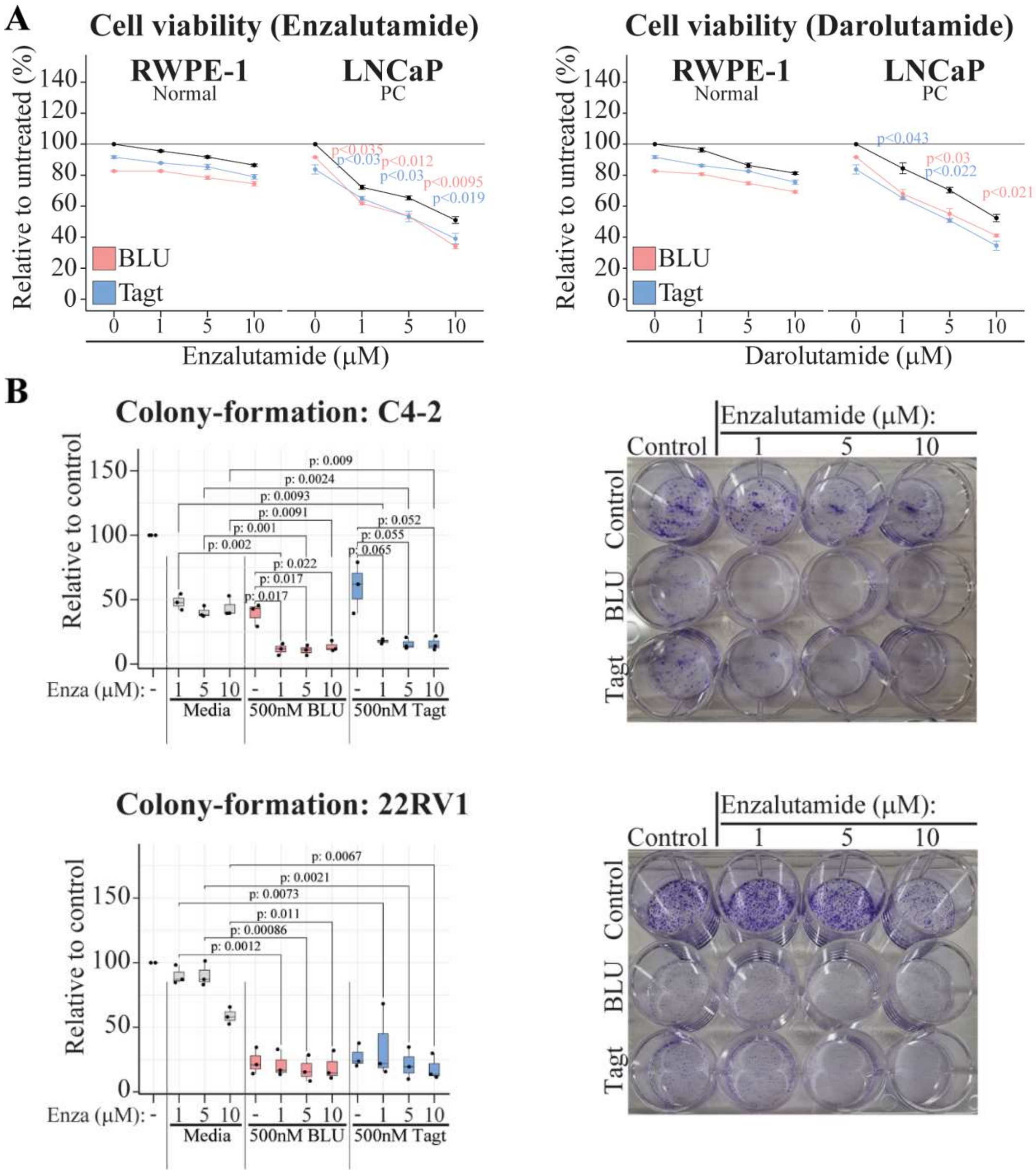

Supplementary figure 11. Combined inhibition of CDK2 and androgen receptor induces anti-proliferative effects on prostate cancer cells. A) Prostate cancer cells (LNCaP) and cell line derived from normal prostate epithelia (RWPE-1) were treated for 4 days as indicated (BLU-222: 500 nM and Tagtociclib: 500 nM), viability assay performed and data is the mean of four biological replicates (each biological replicate comprised 2 technical replicates for LNCaP and 3 technical replicates for RWPE-1) with SEM. Paired two-tailed Student's *t*-test was used to evaluate statistical significance, which is indicated in comparison between CDK2 inhibitor-alone and compound of interest-alone to combination and is color-coded according to CDK2 inhibitor used. **B)** Colony-formation assay. Cells were treated as indicated and colony-formation assay performed after 7 days (3 biological replicates with SEM and paired two-tailed Student's *t*-test was used to assess the significance). Please, refer to figure for the values on significance-calculations.

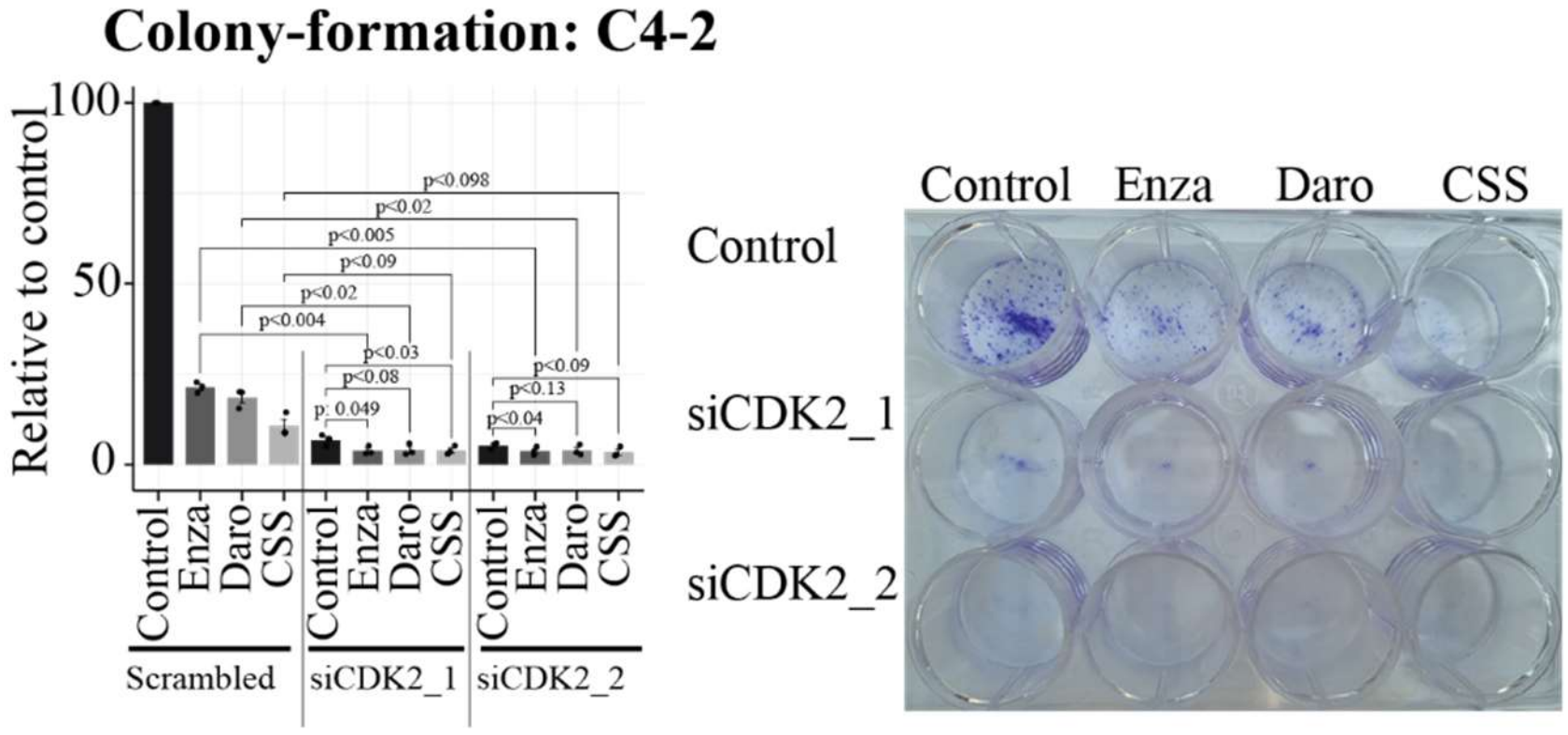

Supplementary figure 12. Knockdown of CDK2 sensitizes castration-resistant prostate cancer cells to anti-androgens. CDK2 was knocked down for a day after which C4-2 cells were treated as indicated and colony-formation assay performed after 7 days (3 biological replicates with SEM and paired samples two-tailed Student's t-test was used to assess the significance). Please, refer to figure for the values on significance-calculations. Darolutamide and Enzalutamide dose: 5μM, CSS= androgen starvation (charcoal-stripped serum).

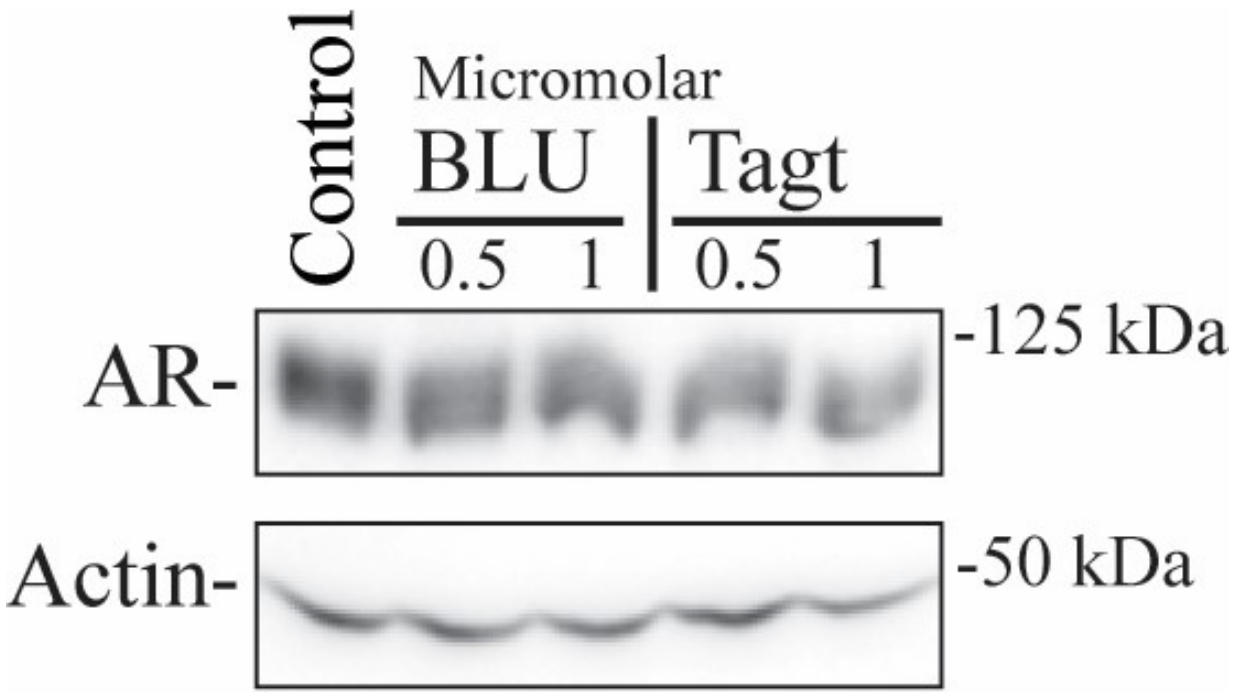

Supplementary figure 13. CDK2 inhibition does not alter AR protein levels after 24 hours treatment. C4-2 cells were treated as indicated for 24 hours and samples analyzed using western blot. Please, note that these are the same samples as those in main figure 2C and actin western blot is the same as in main figure 2C. Data is representative of two biological replicates.

References

1. Taylor BS, Schultz N, Hieronymus H, Gopalan A, Xiao Y, Carver BS, et al. Integrative genomic profiling of human prostate cancer. Cancer Cell. 2010;18(1):11-22.
2. Abida W, Cyrta J, Heller G, Prandi D, Armenia J, Coleman I, et al. Genomic correlates of clinical outcome in advanced prostate cancer. Proc Natl Acad Sci U S A. 2019;116(23):11428-36.
